# Long-term analysis of infections and associated risk factors in patients with multiple sclerosis treated with ocrelizumab: pooled analysis of 13 interventional clinical trials

**DOI:** 10.1177/17562864241277736

**Published:** 2024-10-08

**Authors:** Tobias Derfuss, Robert Bermel, Chien-Ju Lin, Stephen L. Hauser, Ludwig Kappos, Timothy Vollmer, Giancarlo Comi, Gavin Giovannoni, Hans-Peter Hartung, Martin S. Weber, Jianmei Wang, Nikki Jessop, Cathy Chognot, Licinio Craveiro, Amit Bar-Or

**Affiliations:** Department of Neurology, University Hospital Basel, University of Basel, Hebelstrasse 20, Basel 4031, Switzerland; Mellen Center for MS, Cleveland Clinic, Cleveland, OH, USA; Roche Products Ltd, Welwyn Garden City, UK; UCSF Weill Institute for Neurosciences, University of California San Francisco, San Francisco, CA, USA; Research Centre for Clinical Neuroimmunology and Neuroscience Basel, University Hospital Basel, University of Basel, Basel, Switzerland; Department of Neurology, University of Colorado Anschutz Medical Campus, Aurora, CO, USA; Vita-Salute San Raffaele University and Casa di Cura del Policlinico, Milan, Italy; Blizard Institute, Barts and The London School of Medicine and Dentistry, Queen Mary University of London, London, UK; Department of Neurology, UKD, Heinrich-Heine University Düsseldorf, Düsseldorf, Germany; Brain and Mind Centre, University of Sydney, Sydney, Australia; Institute of Neuropathology, University Medical Centre, Göttingen, Germany; Fraunhofer-Institute for Translational Medicine and Pharmacology ITMP, Göttingen, Germany; Roche Products Ltd, Welwyn Garden City, UK; F. Hoffmann-La Roche Ltd, Basel, Switzerland; F. Hoffmann-La Roche Ltd, Basel, Switzerland; F. Hoffmann-La Roche Ltd, Basel, Switzerland; Department of Neurology and Center for Neuroinflammation and Experimental Therapeutics, Perelman School of Medicine, University of Pennsylvania, Philadelphia, PA, USA

**Keywords:** comorbidities, disease-modifying therapies, infections, long-term data, multiple sclerosis, safety, serious infections

## Abstract

**Background:**

Patients with multiple sclerosis (PwMS) have an increased risk of infections.

**Objectives:**

To characterize incidence, clinical characteristics, outcomes and risk factors of infections, and serious infections (SIs) in ocrelizumab (OCR)-treated PwMS.

**Design:**

*Post-hoc* analysis of pooled data from 6155 patients in 13 clinical trials.

**Methods:**

Descriptive analyses of clinical characteristics and outcomes were reported over ⩽14 years. A Poisson Generalized Estimating Equation model was constructed to examine risk factors in a subgroup of patients with longer exposure to OCR (*n* = 2092).

**Results:**

Over a median (max) treatment period of 3.7 (13.9) years, 420/6155 patients (6.8%) experienced 583 SIs, excluding coronavirus disease 2019. Incidence rates in relapsing multiple sclerosis (RMS; 1.50 per 100 patient years [95% confidence interval (CI): 1.34–1.68]) and progressive multiple sclerosis (PMS; 3.70 [95% CI: 3.27–4.17]) remained stable over this period. Lower respiratory tract, urinary tract, abdominal and gastrointestinal, and skin infections were the most commonly reported SIs. Most SIs (~90%) resolved, and treatment with OCR was continued in >80% of cases. The presence of 1 or ⩾2 comorbidities (rate ratio = 1.66, 2.73, respectively), recent relapse activity (2.06), and Expanded Disability Status Scale (EDSS) score ⩾6.0 (2.02) were significant risk factors for SIs in patients with RMS treated over a median (max) period of 8.3 (11.2) years. In patients with primary PMS treated over a median (max) period of 7.1 (11.8) years, an EDSS score ⩾6.0 was associated with the greatest risk of SIs, a 4-fold increase (rate ratio, 4.31), followed by abnormal immunoglobulin (Ig)M levels (1.89), the presence of ⩾2 comorbidities (1.80), and having overweight/obesity (1.46). Time on OCR and abnormal IgG levels were not significantly associated with an increased SI risk.

**Conclusion:**

Continuous long-term treatment with OCR is associated with a manageable infection risk profile. Optimal disease control and addressing modifiable risk factors may reduce the risk of infections.

## Plain language summary

Is continued treatment with ocrelizumab associated with a higher risk of infections and serious infections in patients with multiple sclerosis?

Patients with multiple sclerosis (PwMS) are at an increased risk of infections compared with the general population. Infections are also among the most frequently reported side effect in PwMS treated with ocrelizumab. Initial analyses have shown that serious infection (SI) rates in PwMS treated with ocrelizumab were stable over 6 years, but there is concern that this rate may increase with continued treatment. This study aimed to describe infections and SIs in PwMS treated with ocrelizumab and look into factors that increase patients’ susceptibility to infections.

We analyzed the largest population of PwMS ever treated with ocrelizumab, including 6155 patients from 13 clinical trials. Some of these patients received ocrelizumab for as long as 14 years. Approximately 7 out of every 100 patients experienced SIs, excluding COVID-19 infections, with no increase in yearly rates of SIs over time. Pneumonia and urinary tract infections were the most common SIs. Almost all patients recovered from their infections (>9 out of 10 cases), and continued treatment with ocrelizumab (>8 out of 10 cases).

When looking at factors that made PwMS more prone to SIs, we found that patients with relapsing MS had an increased risk if they had experienced recent MS relapses, severe walking difficulties, or other health conditions like diabetes and bladder problems. Patients with primary progressive MS (PPMS) with severe walking difficulties were four times more likely to have SIs. Having other health conditions like heart or bladder problems, low levels of immunoglobulin M, or excess weight also increased the risk of SIs in patients with PPMS.

In conclusion, continued treatment with ocrelizumab did not increase the risk of SIs, and most of those infections resolved without stopping ocrelizumab treatment. Addressing certain health conditions and achieving a good control of the MS disease may help to reduce the risk of SIs.

## Introduction

Real-world studies have shown that patients with multiple sclerosis (PwMS) have an increased risk of infections and infection-related hospitalizations compared with non-multiple sclerosis (MS) controls.^1–6^ Infections are one of the most commonly reported comorbidities in PwMS, even in the prodromal phase of MS,^6,7^ and are a frequent cause of mortality, driven mostly by respiratory and urinary tract infections (UTIs).^8,9^ In patients treated with ocrelizumab (OCR), infections are among the most frequently reported adverse events (AEs), while serious infections (SIs) are infrequent and their incidence has generally been stable over time.^10^ Importantly, characteristics of all infections in clinical trials appear consistent with infection-related hospitalizations reported in real-world cohorts.^1–6,10^ Still, there is a putative concern that continuous, long-term treatment with OCR and other anti-CD20s, including rituximab, ofatumumab, and ublituximab may increase the risk of infections,^11–14^ partly related to the apparent association between SI rates and decreased levels of immunoglobulin (Ig)G, even though this association is reported in <1% of OCR-treated PwMS.^10^

Understanding the factors that drive the risk of SIs is fundamental for the effective management of OCR-treated patients, especially since most are expected to receive treatment over many years. Long-term extensions of interventional clinical trials provide high-quality datasets and offer a unique opportunity to investigate potential risk factors. Here, we aim to describe the frequency, clinical characteristics, and outcomes of infections and SIs in PwMS continuously treated with OCR across 13 clinical trials, and to characterize the risk factors associated with infections and SIs.

## Methods

### Studies and data collection period

All PwMS who received at least one dose of OCR during the controlled treatment period in one phase II clinical trial, three phase III pivotal clinical trials (OPERA I, OPERA II, and ORATORIO), and respective open-label extension (OLE) periods (OLERO) phase IIIb study, seven phase III, and one phase IV clinical trials were included in this analysis. A summary of study design, study populations, and total exposure can be found in Supplemental Table 1. Briefly, PwMS received OCR 600 mg by intravenous infusion every 24 weeks (6 months). Data collection spanned a period of about 14 years, from 2008 until the clinical cut-off date of November 25, 2022; although patient numbers beyond Year 11 of treatment were limited.

### Definitions of outcomes

Infections and SIs were defined using AEs coded as per Medical Dictionary for Regulatory Activities (MedDRA, versions 18.0 to 23.1) System Organ Class “Infections and Infestations.”^15^ Standard criteria for determining seriousness and severity were used (Supplemental Table 2).^16,17^ Unless stated, data are reported excluding confirmed or suspected (symptomatic) coronavirus disease 2019 (COVID-19) cases to allow for a long-term analysis not confounded by the COVID-19 pandemic. Suspected COVID-19 cases were identified through reported terms.^15^

### Risk factors

Analyses of risk factors for all infections and SIs were performed in phase III populations (OPERA and ORATORIO; *n* = 2092 patients) as these included patients with the longest OCR exposure. Analyses included data up to the end of these two studies (December 2022). Demographic risk factors considered were age, sex, body mass index (BMI), and geographic location. Comorbidities of relevance for infections were selected according to an adapted version of the Charlson–Deyo Comorbidity Index^18^ and the Elixhauser Comorbidity Index^19^ (Supplemental Table 3). Disease characteristics included in the model were disease duration, Expanded Disability Status Scale (EDSS) score, prior disease-modifying therapy (DMT), and occurrence of relapses (for relapsing MS [RMS]). Absolute lymphocyte count, neutrophil count, IgM, IgG, and B-cell levels (measured at least every 24 weeks) were also included as potential risk factors. Since the analysis also included patients who switched from comparators (interferon β-1a [OPERA] or placebo [ORATORIO]) to OCR, the original randomization arm and time on OCR were included.

### Statistical analysis

Cohort characteristics were summarized using mean, median, and ranges for continuous variables, and frequencies (%) for categorical variables. Exposure time included both time on OCR treatment and subsequent off-treatment safety follow-up. To account for the different exposure lengths, the rate of infections and SIs per 100 patient years (PY) are presented, with 95% confidence intervals (CIs) based on a Poisson distribution.

Mean IgG and IgM levels over a period of up to 10 years were analyzed as trajectories according to baseline quartile values. To predict IgG and IgM trajectories beyond 10 years, the observed data were fitted by an exponential or a linear decay model. The model that best explained the trajectories was selected using the Akaike Information Criterion and the residual sums of squares.

**Figure 1.**
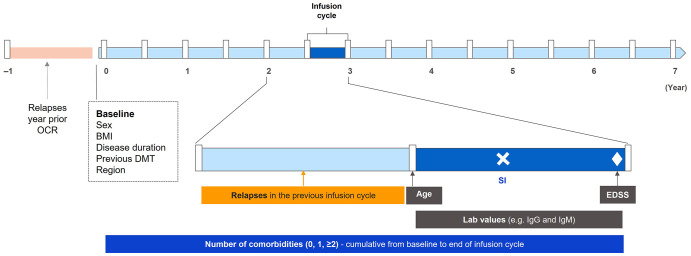
Construction and interpretation of covariates in the Poisson Generalized Estimating Equation regression model. A Poisson Generalized Estimating Equation regression model with repeated measurements over multiple infusion and noninfusion 180-day cycles was used (noninfusion cycles correspond to periods in the safety follow-up, which are counted from the last infusion until the last known follow-up). In this example, a SI (white cross) is reported during infusion cycle 6. Covariates considered at baseline are sex, BMI, region of origin, previous DMT, relapses in year prior to OCR start (for patients switching from interferon or placebo, the year before OCR start corresponds to the last year of the controlled treatment period), disease duration, and study arm. Time-dependent covariates include (1) age at the beginning of each infusion cycle, (2) clinical relapses during the previous infusion cycle, (3) EDSS nearest to the end of the infusion cycle (EDSS values at unscheduled visits and within up to 30 days after the onset of a protocol-defined relapse to confirm progression are excluded), (4) cumulative comorbidities from baseline to the end of the infusion cycle (with the exception of previous infections, which are considered only before OCR start), and (5) laboratory assessments: IgG, IgM, and lymphocytes. As there are multiple measurements over an infusion cycle, a weighted value was used, calculated as follows: ⅀[lab value_*i* × weight_*i*]/180 days. This allows for a representative value to be obtained for an infusion cycle, where weight_*i* is the duration between assessments within the infusion cycle (using the start or end of the infusion cycle as the boundary for the assessment period). BMI, body mass index; DMT, disease-modifying therapy; EDSS, Expanded Disability Status Scale; Ig, immunoglobulin; OCR, ocrelizumab; SI, serious infection.

In order to examine risk factors for all infections and SIs, a Poisson Generalized Estimating Equation multivariate model with repeated measurements was constructed (Figure 1). Separate models were built for RMS and primary progressive MS (PPMS). The selection of variables including their time dependency (baseline or time-varying) was done using univariate analyses. Their respective formats (continuous vs categorical) were assessed using frequency tables and rates of infections and SIs (Supplemental Tables 4 and 5). Through this step, neutrophil and B-cell levels were excluded as covariates due to low numbers of associated SIs to avoid the consequent risk of sparse data bias.^20^ Finally, covariates with a significance level *p* ⩽ 0.2 in the univariate models were included in the multivariate models.

## Results

### Population

As of November 2022, 6155 patients (RMS = 4558; PMS = 1597) had received OCR in 13 clinical trials (Supplemental Table 1), with 3677 patients (59.7%) and 1407 patients (22.9%) receiving OCR for more than 4 and 8 years, respectively. Patient disposition and reasons for discontinuation can be found in Supplemental Figure 1. Demographic and clinical characteristics at baseline and last known follow-up for all patients and the subset of patients (phase III trials, *n* = 2092 patients) included in the risk factor analysis are shown in Table 1.

**Table 1.** Demographic and clinical characteristics at baseline and/or last known follow-up.^a^

Characteristic, *n* (%) except where specified	All trials (*N* = 6155)		Phase III trials (*N* = 2092)	
	RMS (*n* = 4558)	PMS (*n* = 1597)^b^	OPERA (*N* = 1448)	ORATORIO (*N* = 644)
**Patient years**	21,079.9	7189.5	10,808.6	4713.1
**Median time on treatment, years (range)** ^c^	3.7 (0.0–13.9)	3.7 (0.0–11.6)	8.3 (0.0–11.2)	7.1 (0.1–11.8)
⩽5 years	3207 (70.4)	1119 (70.1)	306 (21.1)	168 (26.1)
5–10 years	1019 (22.4)	278 (17.4)	894 (61.7)	276 (42.9)
>10 years	332 (7.3)	200 (12.5)	248 (17.1)	200 (31.1)
**Mean age at baseline, years (SD)**	35.9 (9.4)	47.9 (9.0)	38.1 (9.3)	45.8 (8.1)
**Mean age at last known follow-up, years (SD)**	40.5 (10.1)	52.5 (8.9)	47.6 (9.2)	55.6 (7.8)
<40 years	2213 (48.6)	144 (9.0)	326 (22.5)	26 (4.0)
40–59 years	2189 (48.0)	1065 (66.7)	945 (65.3)	366 (56.8)
⩾60 years	156 (3.4)	388 (24.3)	177 (12.2)	252 (39.1)
**Sex**				
Female	3018 (66.2)	839 (52.5)	949 (65.5)	320 (49.7)
Male	1540 (33.8)	758 (47.5)	499 (34.5)	324 (50.3)
**Mean BMI at baseline, kg/m^2^ (SD)**	26.5 (6.3)	25.3 (5.2)	26.2 (6.1)	25.0 (4.9)
Underweight <18.5	169 (3.7)	65 (4.1)	56 (3.9)	27 (4.2)
Normal weight 18.5–25.0	2068 (45.4)	818 (51.2)	677 (46.8)	345 (53.6)
Overweight 25.0–30.0	1202 (26.4)	443 (27.7)	389 (26.9)	174 (27.0)
Obese ⩾30.0	1078 (23.7)	254 (15.9)	312 (21.5)	97 (15.1)
Unknown	41 (0.9)	17 (1.1)	14 (1.0)	1 (0.2)
**Subregion**				
ROW^d^	3120 (68.5)	1398 (87.5)	1089 (75.2)	558 (86.6)
USA	1438 (31.5)	199 (12.5)	359 (24.8)	86 (13.4)
**Ethnicity**				
White	3891 (85.4)	1305 (81.7)	1316 (90.9)	606 (94.1)
Others	485 (10.6)	113 (7.1)	132 (9.1)	37 (5.7)
Unknown	182 (4.0)	179 (11.2)	0 (0.0)	1 (0.2)
**Comorbidities at last known follow-up**				
Depression	773 (17.0)	336 (21.0)	432 (29.8)	191 (29.7)
Previous infections^e^	686 (15.1)	256 (16.0)	482 (33.3)	223 (34.6)
Cardiovascular disorders	448 (9.8)	349 (21.9)	270 (18.6)	181 (28.1)
Renal and urinary tract disorders	476 (10.4)	293 (18.3)	253 (17.5)	172 (26.7)
Chronic pulmonary disorders	226 (5.0)	75 (4.7)	125 (8.6)	45 (7.0)
Diabetes	96 (2.1)	78 (4.9)	49 (3.4)	39 (6.1)
Rheumatologic and autoimmune disorders	114 (2.5)	57 (3.6)	50 (3.5)	34 (5.3)
Gastrointestinal disorders	83 (1.8)	46 (2.9)	51 (3.5)	28 (4.3)
Cerebrovascular disorders	6 (0.1)	7 (0.4)	4 (0.3)	4 (0.6)
Malignancies	5 (0.1)	3 (0.2)	4 (0.3)	3 (0.5)
**Number of Comorbidities at last known follow-up**				
0	2588 (56.8)	702 (44.0)	491 (33.9)	258 (40.1)
1	1305 (28.6)	489 (30.6)	458 (31.6)	198 (30.7)
⩾2	665 (14.6)	406 (25.4)	499 (34.5)	188 (29.2)
**Median EDSS at OCR**^f^ start, (IQR)^g^	2.0 (1.5–3.0)	5.0 (4.0–6.0)	2.5 (1.8–3.5)	4.5 (3.5–6.0)
<3.0	2964 (66.5)	79 (4.9)	810 (55.9)	12 (1.9)
3.0–6.0	1433 (32.2)	890 (55.7)	625 (43.2)	529 (82.1)
⩾6.0	59 (1.3)	628 (39.3)	13 (0.9)	102 (15.8)
Unknown	−	–	0 (0.0)	1 (0.2)
**Disease duration since symptom onset, years**				
<5 years	2946 (64.9)	450 (28.7)	658 (45.4)	190 (29.5)
5–10 years	1042 (23.0)	520 (33.1)	405 (28.0)	288 (44.7)
>10 years	550 (12.1)	600 (38.2)	385 (26.6)	147 (22.8)
**Relapses in year before study start** ^h^				
Any relapses	3807 (83.5)	85 (5.3)	1394 (96.3)	–
Mean number of relapses, (SD)	1.18 (0.82)	0.07 (0.30)	1.32 (0.69)	–
**Previous DMTs**				
0	2675 (58.7)	908 (56.9)	1065 (73.5)	571 (88.7)
1	1213 (26.6)	350 (21.9)	345 (23.8)	56 (8.7)
⩾2	670 (14.7)	339 (21.2)	38 (2.6)	17 (2.6)
**Last DMT** ^i^				
Interferons	645 (34.3)	175 (25.4)	252 (65.8)	48 (64.0)
Glatiramer acetate	467 (24.8)	95 (13.8)	118 (30.8)	23 (30.7)
Dimethyl fumarate	357 (19.0)	82 (11.9)	1 (0.3)	–
Fingolimod	228 (12.1)	82 (11.9)	2 (0.5)	–
Natalizumab	13 (0.7)	59 (8.6)	1 (0.3)	1 (1.3)
Biotin	28 (1.5)	39 (5.7)	4 (1.0)	2 (2.7)
Teriflunomide	125 (6.6)	60 (8.7)	–	–
Azathioprine	3 (0.2)	21 (3.0)	1 (0.3)	1 (1.3)
Cyclophosphamide	1 (<0.1)	21 (3.0)	–	–
Other^j^	16 (0.8)	55 (8.0)	4 (1.0)	–

Patients from phase III trials OPERA and ORATORIO are a subset of the entire RMS and PMS population represented in the first two columns. These are the largest cohorts available with longer exposure to OCR. Not all data on BMI, EDSS at OCR start, and disease duration were available for analysis.

a Last known follow-up was at time of study discontinuation/completion or as of November 25, 2022 for patients still ongoing.

b Includes patients with primary progressive MS (*n* = 1076) and secondary progressive MS (*n* = 521).

c Time on OCR of “0” years relates to patients who discontinued after the first infusion.

d Europe, Middle East, Africa, Central and South America, Canada, Australia.

e Previous infection recorded in the medical history before OCR start.

f Last valid assessment before OCR treatment in the CTP and in the open-label extension, for patients originally randomized to OCR and for switchers, respectively.

g For the IQR, 25th and 75th quartiles are reported.

h Not applicable for patients from ORATORIO as this was an exclusion criterion.

i Last DMT refers to that at study start.

j Other: Cladribine, diroximel fumarate, mitoxantrone, methotrexate, laquinimod, daclizumab, masitinib, siponimod, minocycline, OCR, and rituximab.

BMI, body mass index; CTP, controlled treatment period; DMT, disease-modifying therapy; EDSS, Expanded Disability Status Scale; IQR, interquartile range; MS, multiple sclerosis; OCR, ocrelizumab; PMS, progressive multiple sclerosis; RMS, relapsing multiple sclerosis; ROW, rest of the world; SD, standard deviation.

### All infections

#### Rates of all infections

Over a period of up to 14 years, cumulative rates of infections of 66.22 per 100 PY (95% CI: 65.13–67.33) and 61.60 per 100 PY (95% CI: 59.80–63.45) were reported in patients with RMS and PMS, respectively. The yearly incidence rate of infections remained overall stable during this period (Supplemental Table 6).

#### Types of all infections

The three most common types of infections were nasopharyngitis (RMS: 11.23 per 100 PY [95% CI: 10.79–11.70]; PMS: 8.22 [95% CI: 7.57–8.91]), UTIs (RMS: 9.59 [95% CI: 9.18–10.02]; PMS: 18.38 [95% CI: 17.41–19.41]), and upper respiratory tract infections (RMS: 8.97 [95% CI: 8.57–9.38]; PMS: 3.96 [95% CI: 3.52–4.45]).

#### Outcomes and action taken with OCR

**Figure 2.**
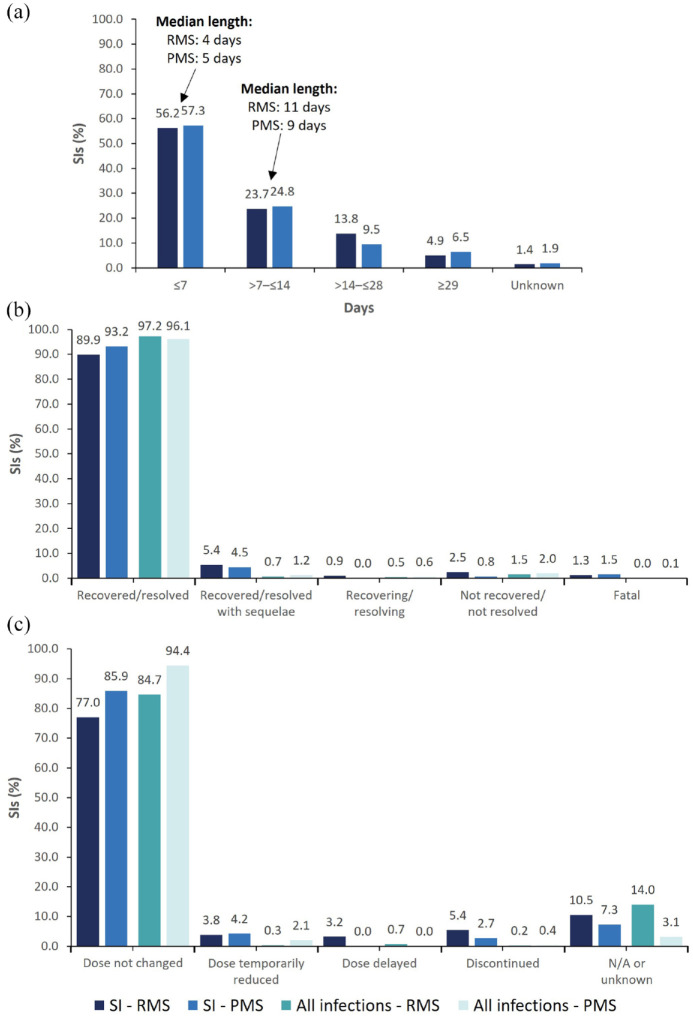
Outcomes and action taken with OCR following all infections and SIs, excluding COVID-19. (a) Length of hospitalization for SIs requiring admission to hospital (*n* = 545/583) in patients with RMS and PMS. (b) Outcomes of infections in patients with RMS and PMS; for SIs, fatal cases include pneumonia (*n* = 2), sepsis (*n* = 2), encephalitis (*n* = 1), enterococcal infection (*n* = 1), pneumonia aspiration (*n* = 1), and urosepsis (*n* = 1). (c) Action taken with OCR following infections in patients with RMS and PMS. CCOD, November 2022. CCOD, clinical cut-off date; COVID-19, coronavirus disease 2019; N/A, not applicable; OCR, ocrelizumab; PMS, progressive multiple sclerosis; RMS, relapsing multiple sclerosis; SI, serious infection.

The majority of infections were reported as resolved (RMS: 97.2%; PMS: 96.1%), and treatment was discontinued in 1.1% of patients with infections, representing 0.3% (*n* = 47/6155) of all patients (Figure 2).

### Serious infections

#### Rates of SIs

**Figure 3.**
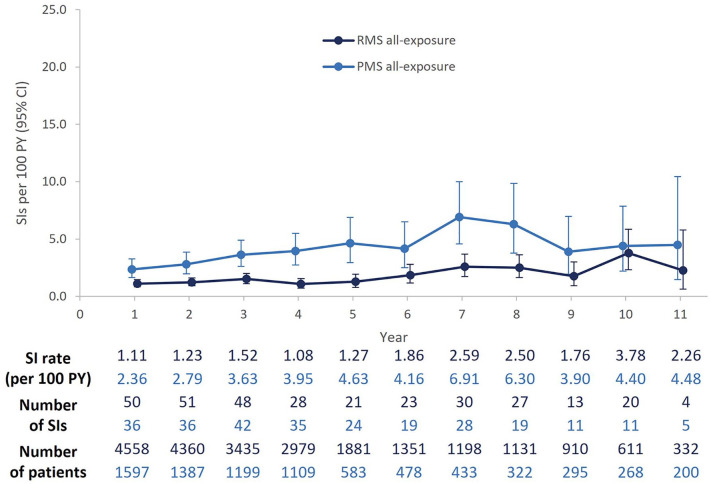
Yearly rates of SIs in RMS and PMS populations, excluding COVID-19. Pooled yearly SI rates from the 13 OCR clinical trials are presented. Patients with longer exposure (⩾6 years) are from the extension periods of phase II and phase III studies, including those originally randomized to the comparators interferon β-1a (OPERA) or placebo (ORATORIO) who switched to open-label OCR treatment. Patients with exposure >11 years (up to 14 years) are not represented in the graph; two SIs were reported in this group of patients beyond 11 years of treatment. CCOD, November 2022. CCOD, clinical cut-off date; CI, confidence interval; COVID-19, coronavirus disease 2019; OCR, ocrelizumab; PMS, progressive multiple sclerosis; PY, patient years; RMS, relapsing multiple sclerosis; SI, serious infection.

Over a period of up to 14 years, 420/6155 patients (6.8%) experienced 583 SIs (RMS, 252/4558 [5.5%]; PMS, 168/1597 [10.5%]); additionally, 262 patients experienced 275 COVID-19 infections. A total of 98 (1.6%) patients experienced >1 SI, of which the majority (66/98) experienced two SIs. UTIs, abdominal infections (mostly gastroenteritis), and skin infections (cellulitis) were the most common repeated SIs. In both RMS and PMS populations, incidence rates fluctuated over time but remained generally stable over the follow-up period (Figure 3). The cumulative rate of SIs was higher in patients with PMS (3.70 per 100 PY [95% CI: 3.27–4.17]) than RMS (1.50 per 100 PY [95% CI: 1.34–1.68]; Supplemental Table 7). A slight increase observed at Year 7 in patients with PMS, compared with Year 6, was driven by a greater number of cases of non-COVID-19 pneumonia. A similar pattern was observed at Year 10 (vs Year 9) for patients with RMS, who experienced additional cases of non-COVID-19 respiratory infections. These two periods largely overlap with most patients being within COVID-19 waves (data not shown). Yearly rates of SIs in both populations including COVID-19 events can be found in Supplemental Figure 2.

#### Types of SIs

##### Most common SIs

As of November 2022, lower respiratory tract infections (LRTIs; *n* = 137, 16.0%, mostly pneumonia), UTIs (*n* = 132, 15.4%), abdominal and gastrointestinal infections (*n* = 78, 9.1%; e.g., appendicitis and gastroenteritis), skin infections (*n* = 64, 7.5%), and sepsis (*n* = 54, 6.3%) were the most commonly reported SIs (Supplemental Table 7). An additional 275 COVID-19-related infections (32.1%) were also reported. Patient demographics, comorbidities, and disease characteristics associated with these most common SIs can be found in Table 2.

**Table 2.** Characteristics of patients with the five most common types of SIs.

Type (number of patients)	Number (%) of patients (except where specified)					
	All patients (*n* = 6155)	UTI (*n* = 103)	LRTI^a^ (*n* = 129)	Abdominal infections (*n* = 53)	Sepsis^b^ (*n* = 47)	Skin infections^c^ (*n* = 43)
**Number of SIs**	**858**	**132**	**137**	**78**	**54**	**64**
Type of MS						
RMS	4558 (74.1)	45 (43.7)	82 (63.6)	31 (58.5)	17 (36.2)	20 (46.5)
PMS	1597 (25.9)	58 (56.3)	47 (36.4)	22 (41.5)	30 (63.8)	23 (53.5)
**Age at the time of first event onset**						
**Mean**, years (SD)	–	46.2 (10.7)	46.8 (9.9)	41.8 (12.0)	48.6 (10.3)	47.5 (9.8)
<40 years	2961 (52.9)^d^	30 (29.1)	36 (27.9)	23 (43.4)	9 (19.1)	12 (27.9)
40–59 years	2761 (44.9)^d^	67 (65.0)	84 (65.1)	28 (52.8)	34 (72.3)	27 (62.8)
⩾60 years	137 (2.2)^d^	6 (5.8)	9 (7.0)	2 (3.8)	4 (8.5)	4 (9.3)
**Sex**						
Female	3857 (62.7)	60 (58.3)	71 (55.0)	30 (56.6)	23 (48.9)	16 (37.2)
Male	2298 (37.3)	43 (41.7)	58 (45.0)	23 (43.4)	24 (51.1)	27 (62.8)
**EDSS score at baseline** ^e^						
**Median (IQR)**	2.5 (1.5–4.0)	4.5 (3.0–6.0)	3.5 (2.0–5.0)	3.5 (2.0–4.0)	5.0 (3.5–6.5)	4.0 (2.5–5.5)
<3.0	3043 (50.3)	17 (16.5)	44 (34.6)	18 (35.3)	9 (20.5)	11 (25.6)
3.0–6.0	2323 (38.4)	53 (51.5)	60 (47.2)	27 (52.9)	18 (40.9)	22 (51.2)
⩾6.0	687 (11.3)	33 (32.0)	23 (18.1)	6 (11.8)	17 (38.6)	10 (23.3)
**FSS at baseline** ^e^						
FSS pyramidal ⩾4	291 (4.8)	12 (11.7)	9 (7.1)	1 (2.0)	7 (15.9)	6 (14.0)
FSS cerebellar ⩾4	5 (<0.1)	0 (0.0)	0 (0.0)	0 (0.0)	0 (0.0)	0 (0.0)
FSS brainstem ⩾3	159 (2.6)	2 (1.9)	4 (3.1)	2 (3.9)	3 (6.8)	2 (4.7)
FSS sensory ⩾4	59 (1.0)	0 (0.0)	0 (0.0)	2 (3.9)	1 (2.3)	1 (2.3)
FSS bowel and bladder ⩾3	233 (3.8)	16 (15.5)	15 (11.8)	2 (3.9)	5 (11.4)	6 (14.0)
FSS cerebral ⩾4	93 (1.5)	5 (4.9)	6 (4.7)	0 (0.0)	4 (9.3)	2 (4.7)
FSS visual ⩾5	197 (3.3)	8 (7.8)	12 (9.4)	1 (2.0)	3 (6.8)	2 (4.7)
Ambulation score ⩾6	601 (10.3)	32 (32.7)	22 (18.3)	5 (10.6)	17 (41.5)	10 (23.8)
**BMI at baseline**						
**Mean, kg/m^2^ (SD)**	26.2 (6.0)	25.7 (6.4)	26.2 (7.0)	26.4 (5.5)	27.8 (6.9)	27.6 (7.6)
Underweight <18.5	234 (3.8)	6 (5.8)	5 (3.9)	1 (2.0)	3 (6.4)	2 (4.7)
Normal weight 18.5–25.0	2893 (47.4)	52 (50.5)	64 (50.0)	22 (43.1)	12 (25.5)	15 (34.9)
Overweight >25.0	2970 (48.7)	45 (43.7)	59 (46.1)	28 (54.9)	32 (68.1)	26 (60.5)
**Relapses in year before baseline**	3892 (63.3)	39 (37.9)	81 (62.8)	29 (54.7)	13 (27.7)	19 (44.2)
**Previous DMTs**						
0	3583 (58.2)	68 (66.0)	98 (76.0)	34 (64.2)	34 (72.3)	24 (55.8)
1	1563 (25.4)	24 (23.3)	26 (20.2)	14 (26.4)	12 (25.5)	13 (30.2)
⩾2	1009 (16.4)	11 (10.7)	5 (3.9)	5 (9.4)	1 (2.1)	6 (14.0)
**Comorbidities at baseline** ^f^						
Previous infections	942 (15.3)	26 (25.2)	24 (18.6)	9 (17.0)	9 (19.1)	9 (20.9)
Depression	687 (11.2)	15 (14.6)	30 (23.3)	9 (17.0)	19 (40.4)	10 (23.3)
Cardiovascular	519 (8.4)	13 (12.6)	15 (11.6)	7 (13.2)	12 (25.5)	6 (14.0)
Urinary tract	300 (4.9)	10 (9.7)	16 (12.4)	4 (7.5)	8 (17.0)	5 (11.6)
Diabetes	120 (1.9)	3 (2.9)	5 (3.9)	2 (3.8)	4 (8.5)	6 (14.0)
Chronic pulmonary	180 (2.9)	0 (0.0)	5 (3.9)	2 (3.8)	1 (2.1)	3 (7.0)
Rheumatologic and autoimmune	66 (1.1)	3 (2.9)	1 (0.8)	2 (3.8)	0 (0.0)	1 (2.3)
Gastrointestinal	53 (0.9)	0 (0.0)	0 (0.0)	0 (0.0)	0 (0.0)	1 (2.3)
Cerebrovascular	9 (0.1)	1 (1.0)	2 (1.6)	0 (0.0)	0 (0.0)	0 (0.0)
**Number of comorbidities at last known follow-up**						
0	3290 (53.5)	34 (33.0)	34 (26.4)	19 (35.8)	6 (12.8)	8 (18.6)
1	1794 (29.1)	34 (33.0)	42 (32.6)	18 (34.0)	11 (23.4)	12 (27.9)
⩾2	1071 (17.4)	35 (34.0)	53 (41.1)	16 (30.2)	30 (63.8)	23 (53.5)

Statistics are *n* (%) unless otherwise stated (shaded rows represent mean [SD]).

a COVID-19 cases excluded.

b The preferred term, “UROSEPSIS,” is included in the “Sepsis” column and excluded from the “UTI” column. Urosepsis was the most common type of sepsis (*n* = 18/54 events).

c Cellulitis (of the lower extremities) was the most common type of serious skin infection (data not shown).

d “Age at time of first event onset” is not available for “All patients,” as such, baseline age has been presented.

e EDSS/FSS baseline defined as the last valid assessment before treatment; unconverted functional scores are presented.

f The total number of patients (not events) is used as the denominator for percentages. Note that one patient might have multiple comorbidities. Investigator text for AEs encoded using MedDRA version 23.1. Multiple occurrences of the same AE in one patient will be counted multiple times. SIs are defined using AEs falling into the MedDRA System Organ Class “Infections and Infestations” and using “Is the event non-serious or serious” from the AEs case report form page.

AE, adverse event; BMI, body mass index; COVID-19, coronavirus disease 2019; DMT, disease-modifying therapy; EDSS, Expanded Disability Status Scale; FSS, Functional Systems Scores; IQR, interquartile range; LRTI, lower respiratory tract infection; MedDRA, Medical Dictionary for Regulatory Activities; MS, multiple sclerosis; PMS, progressive multiple sclerosis; RMS, relapsing multiple sclerosis; SD, standard deviation; SI, serious infection; UTI, urinary tract infection.

##### Hepatitis

No cases of hepatitis B (HBV) de novo infection were observed, and no HBV reactivation (clinical or laboratory) was reported among 175/6155 (2.8%) of patients with a positive HBV core antibody (HBcAb) titer at baseline (patients at risk of reactivation). A total of 12/175 patients were receiving prophylactic antiviral treatment (entecavir, lamivudine, or tenofovir) at the time of enrollment and continued on treatment over the duration of the studies. One case of fulminant echovirus 25-associated hepatitis was reported in a patient with RMS in OPERA.^21^

##### Herpes and opportunistic infections

Serious herpes virus-associated infections were uncommon (*n* = 9, 0.03 per 100 PY [95% CI: 0.02–0.06]; Supplemental Table 7 for more details). Seven cases were hospitalized, but none were life-threatening. All nine cases resolved (one with sequelae), and OCR dose remained unchanged in six cases (two were delayed and one discontinued).

No cases of progressive multifocal leukoencephalopathy, fever of unknown origin, cryptococcosis, aspergillosis, listeriosis, toxoplasmosis, cytomegalovirus, and serious human papilloma virus were reported in the clinical trial population.

##### Central nervous system infections

Serious central nervous system (CNS) infections were uncommon (*n* = 13, 0.05 per 100 PY [95% CI: 0.02–0.08]) and included five cases of encephalitis, seven cases of meningitis (three viral, two bacterial, one unknown, and one aseptic), and one case of neuroborreliosis. Most (*n* = 9/13) resolved with minor or no residual neurological deficits; two were reported as “not recovered/not resolved”; and one was fatal. Additional case details can be found in Supplemental Table 8. The incidence rate of serious CNS infections is consistent with rates observed in two large real-world MS cohorts (0.04–0.05 per 100 PY).^6,22^

#### Hospitalization, outcomes, and action taken with OCR

Hospitalization was reported for 93.5% of SIs (*n* = 545/583) and was more frequent in patients with PMS (98.5%) compared with those with RMS (89.3%); among hospitalized patients with either RMS or PMS, the length of hospitalization was ≤ 7 days in 56.7% of cases (Figure 2(a)). The majority of SIs were reported as recovered/resolved (Figure 2(b)). Excluding COVID-19 fatalities (*n* = 48), there were eight infection-related fatalities were reported (pneumonia, *n* = 3; sepsis, *n* = 2; encephalitis, *n* = 1; enterococcal infection, *n* = 1; urosepsis, *n* = 1). Treatment was discontinued in 4.2% of patients with SIs, representing 0.4% (*n* = 24/6155) of all patients (Figure 2(c)). Length of hospitalization, outcomes, and action taken with OCR for SIs, including COVID-19 events, can be found in Supplemental Figure 3.

### Risk factors for infections and SIs

The exploration of risk factors potentially associated with all infections, and more specifically with SIs, was conducted in the subset of patients enrolled into the pivotal phase III trials and associated long-term OLEs (*n* = 2092), with a median (max) time on the treatment of 8.3 (11.2) and 7.1 (11.8) years for patients with RMS and PPMS, respectively. Mean (standard deviation) age at the last known follow-up was 45.5 (9.8) years for RMS and 53.2 (8.4) years for PPMS. For more details on demographics and disease characteristics, see Table 1 (columns OPERA and ORATORIO).

#### Immunoglobulins

**Figure 4.**
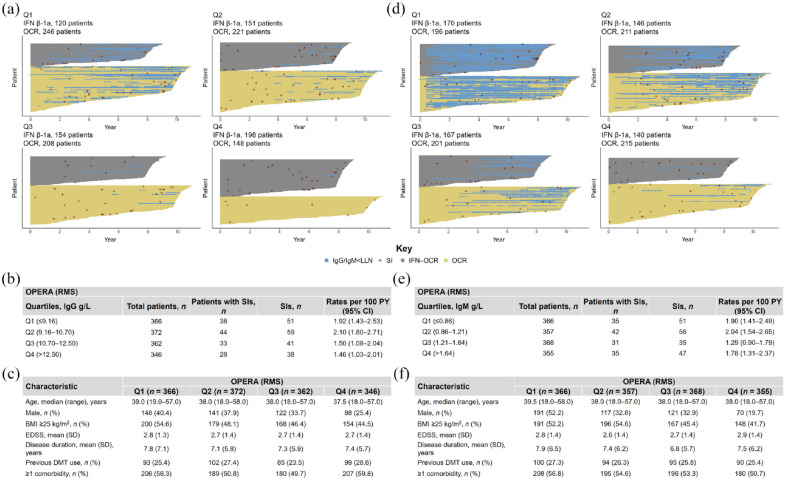
Periods of IgG and IgM below LLN across baseline quartiles and association with SIs in OPERA (excluding COVID-19). Results for (a–c) IgG and (d–f) IgM quartiles. (a, d) Each horizontal yellow line represents OCR exposure of individual patients who were initially randomized to OCR. Each gray line represents OCR exposure of individual patients who were initially randomized to IFN and then switched to OCR. Blue horizontal lines represent the periods where Ig levels dropped below LLN (IgG LLN = 5.65 g/L; IgM LLN = 0.4 g/L). Each red cross represents an SI. Number of patients with SIs and cumulative rates of SIs per (b) baseline IgG quartiles and (e) IgM quartiles. Baseline demographics and disease characteristics of patients by (c) baseline IgG quartiles and (f) baseline IgM quartiles. CCOD, November 2022. BMI, body mass index; CCOD, clinical cut-off date; CI, confidence interval; DMT, disease-modifying therapy; EDSS, Expanded Disability Status Scale; IFN, interferon; Ig, immunoglobulin; LLN, lower limit of normal; OCR, ocrelizumab; PY, patient years; Q, quartile; RMS, relapsing multiple sclerosis; SD, standard deviation; SI, serious infection.

**Figure 5.**
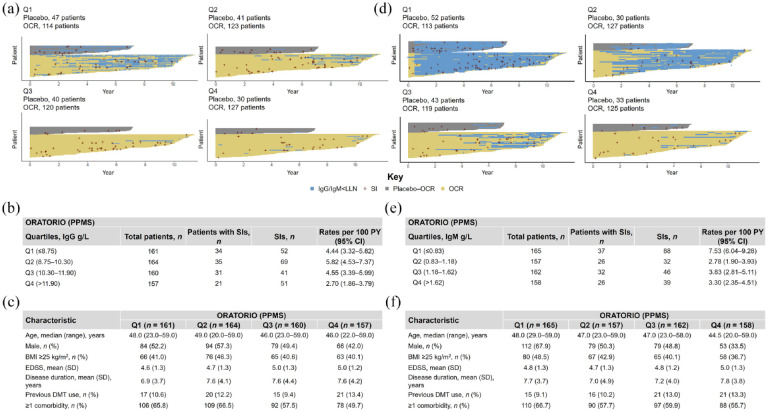
Periods of IgG and IgM below LLN across baseline quartiles and association with SIs in ORATORIO (excluding COVID-19). Results for (a–c) IgG and (d–f) IgM quartiles. (a, d) Each horizontal yellow line represents OCR exposure of individual patients who were initially randomized to OCR. Each gray line represents OCR exposure of individual patients who were initially randomized to placebo and then switched to OCR. Blue horizontal lines represent the periods where Ig levels dropped below LLN (IgG LLN = 5.65 g/L; IgM LLN = 0.4 g/L). Each red cross represents an SI. Number of patients with SIs and cumulative rates of SIs per (b) baseline IgG quartiles and (e) IgM quartiles. Baseline demographics and disease characteristics of patients by (c) baseline IgG quartiles and (f) baseline IgM quartiles. CCOD, November 2022. BMI, body mass index; CCOD, clinical cut-off date; CI, confidence interval; DMT, disease-modifying therapy; EDSS, Expanded Disability Status Scale; Ig, immunoglobulin; LLN, lower limit of normal; OCR, ocrelizumab; PPMS, primary progressive multiple sclerosis; PY, patient years; Q, quartile; SD, standard deviation; SI, serious infection.

After a period of 12 years of OCR treatment, IgG levels remained above the lower limit of normal (LLN = 5.65 g/L) for the majority of patients (RMS-OPERA: 84.5%; PPMS-ORATORIO: 83.7%); most patients experiencing periods of IgG < LLN (blue lines in Figures 4 and 5) were in the lowest baseline IgG quartile. Among patients with RMS and PPMS who experienced periods of IgG < LLN, IgG levels returned to normal levels within three infusion cycles in 43.3% (*n* = 94/217) and 53.4% (*n* = 47/88) of patients, respectively. The temporal evolution of IgG across the different quartiles followed a pattern that could be better explained by an exponential decay model, suggesting that after an initially marked decrease, IgG levels may reach a plateau in patients receiving long-term OCR treatment (Supplemental Figure 4(A)–(C)). A similar, albeit more accelerated exponential decay in the first 2 years was also observed for IgM (Supplemental Figure 4(D)–(F)).

**Figure 6.**
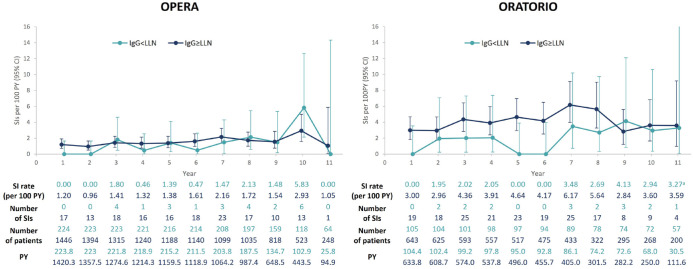
Yearly rates of SIs in patients during periods of IgG < LLN and IgG ⩾ LLN, excluding COVID-19 infections, in patients with RMS (OPERA, *n* = 1488) and PPMS (ORATORIO, *n* = 644). SIs were considered associated with IgG < LLN if the onset of the SI occurred while IgG < LLN. If the onset occurred while IgG levels were in the normal range, even if the SI resolution occurred while IgG < LLN, the infection was not considered to be associated with IgG < LLN. In any given year, a single patient could experience SIs while IgG was normal or below normal; therefore, the same patient can contribute to both curves (this explains why, for example, in Year 1, the sum of patients in both curves is 1670, whereas the number of patients at risk in the same period is 1488). Year 12 data are not represented in the graph as no SIs were reported. CCOD, November 2022. ^a^95% CI at Year 11 for patients with IgG < LLN from ORATORIO: 0.08–18.24. CCOD, clinical cut-off date; CI, confidence interval; COVID-19, coronavirus disease 2019; Ig, immunoglobulin; LLN, lower limit of normal; OCR, ocrelizumab; PPMS, primary progressive multiple sclerosis; PY, patient years; RMS, relapsing multiple sclerosis; SI, serious infection.

Cumulative rates of SIs were broadly similar across the different IgG quartiles both for patients with RMS and PPMS (Figures 4 and 5). Similar rates of SIs were also observed across IgM quartiles, except for patients with PPMS in the lowest IgM baseline quartile (Figures 4 and 5). Yearly rates of SIs during periods of IgG < LLN remained stable, with fluctuations that were consistent with rates of SIs observed in patients with IgG levels at normal levels (Figure 6). Notably, the rate of SIs observed over time in patients experiencing periods of IgG < LLN were not greater than those during IgG > LLN. Types and characteristics of SIs during periods of low IgG were similar to overall SIs, with UTIs and LRTIs being the most commonly reported SIs (Supplemental Table 9). It should be noted that most of these patients showed high comorbidity burden (RMS and PPMS) and, in the case of patients with PPMS, also high disability levels (EDSS ⩾6.0).

Over this period of up to 12 years, a total of 9 of 2092 patients discontinued treatment while their IgG levels were ⩽LLN, of which three discontinuations were associated with SIs, three were associated with non-SIs, and three were not associated with an infection. Excluding COVID-19 (*n* = 6), a total of 22 patients discontinued treatment while their IgM levels were ⩽LLN; 12 associated with SIs, 7 with non-SIs, and 3 not associated with any infections. A total of eight patients who discontinued treatment had both IgG and IgM below LLN.

Intravenous immunoglobulin (IVIg) replacement therapy was reported in *n* = 12/2092 patients, in the context of infections (*n* = 4) or hypogammaglobulinemia (*n* = 8). IVIg was also reported as a treatment for COVID-19 in nine patients.

### Univariate and multivariate analysis

#### Patients with RMS (OPERA)

In the univariate analysis, several covariates were associated with a higher risk of SIs in patients with RMS (Supplemental Figure 5), namely, the presence of comorbidities (⩾2 comorbidities, rate ratio, 3.12 [95% CI: 1.97–4.92]; one comorbidity, 1.87 [95% CI: 1.22–2.88]), a high disability status (EDSS ⩾6.0, 2.94 [95% CI: 1.82–4.74]; EDSS 3.5–5.5, 1.64 [95% CI: 1.10–2.44]), IgG < LLN (2.53 [95% CI: 1.62–3.97]), and recent clinical activity (2.51 [95% CI: 1.47–4.30]). Regarding comorbidities, the highest risk was observed in patients with cerebrovascular disorders (5.03 [95% CI: 1.27–19.86]), diabetes (2.86 [95% CI: 1.30–6.28]), renal and urinary comorbidities (2.58 [95% CI: 1.60–4.14]), followed by rheumatological and autoimmune disorders (2.30 [95% CI: 1.13–4.71]), and previous infections (1.75 [95% CI: 1.19–2.57]). A smaller risk was observed for older age at the start of OCR treatment (>55 years, 1.87 [95% CI: 1.10–3.20]; 40–55 years, 1.49 [95% CI: 1.02–2.18]), a longer disease duration (>10 years, 1.73 [95% CI: 1.14–2.60]), IgM < LLN (1.53 [95% CI: 1.10–2.12]), prior DMT use (1.51 [95% CI: 1.00–2.29]), and a longer treatment duration on OCR (1.09 [95% CI: 1.03–1.15]). Lymphopenia was not associated with an increased risk of SIs (1.42 [95% CI: 0.69–2.92]).

**Figure 7.**
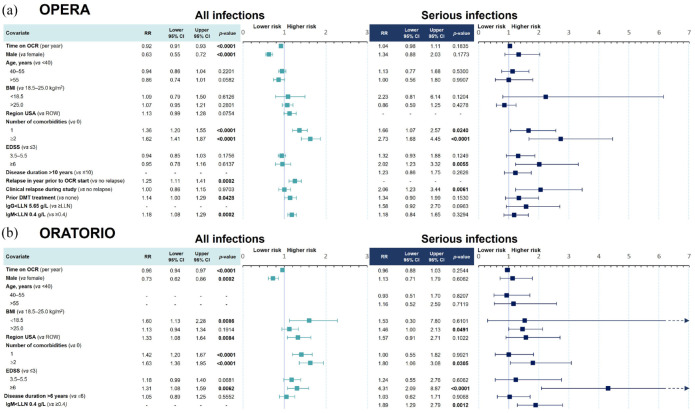
Multivariate model for risk of infections and SIs in (a) OPERA and (b) ORATORIO. (a) Risk of all infections and SIs in OPERA (RMS) over a period of up to 11.2 years. (b) Risk of all infections and SIs in ORATORIO (PPMS), over a period of up to 11.8 years. Significant covariates (*p*-values < 0.05) are marked in bold. Covariates were selected from univariate models if their significance level was *p* ⩽ 0.2. Each model (RMS/PPMS and all infections/SIs) was treated independently, which may result in different covariates being selected. For example, IgG < LLN was not selected for the multivariate analysis of all infections, but it was selected for the multivariate analysis of SIs in patients with RMS in OPERA. Covariates with no results (marked “-”) correspond to covariates not selected from the univariate analysis due to its significance level being *p* > 0.2. CCOD, November 2022. BMI, body mass index; CCOD, clinical cut-off date; CI, confidence interval; DMT, disease-modifying therapy; EDSS, Expanded Disability Status Scale; Ig, immunoglobulin; LLN, lower limit of normal; OCR, ocrelizumab; PPMS, primary progressive multiple sclerosis; RMS, relapsing multiple sclerosis; ROW, rest of the world; RR, rate ratio; SI, serious infection.

In the multivariate model (Figure 7(a)), only the presence of ⩾1 comorbidities (one comorbidity, 1.66 [95% CI: 1.07–2.57]; ⩾2 comorbidities, 2.73 [95% CI: 1.68–4.45]), recent relapse activity (2.06 [95% CI: 1.23–3.44]), and EDSS score ⩾6.0 (2.02 [95% CI: 1.23–3.32]) remained significant independent risk factors for SIs. Hypogammaglobulinemia and longer treatment duration with OCR were not associated with a higher risk of SIs.

The results of both univariate and multivariate models for all infections (serious and nonserious) were overall consistent with the results observed for SIs (Figure 7(a) and Supplemental Figure 5). In the multivariate analysis, having a higher comorbidity burden (⩾2 comorbidities, 1.62 [95% CI: 1.41–1.87]; one comorbidity, 1.36 [95% CI: 1.20–1.55]) and relapse activity before OCR treatment start (1.25 [95% CI: 1.11–1.41]) presented as the highest risk factors for all infections, while previously treated patients (1.14 [95% CI: 1.00–1.29]) and patients with IgM < LLN (1.18 [95% CI: 1.08–1.29]) showed a small increased risk. Male patients also exhibited a reduced risk of infections compared with female patients (0.63 [95% CI: 0.55–0.72]). Time on OCR (0.92 [95% CI: 0.91–0.93]) was not associated with an increased risk of infections.

#### Patients with PPMS (ORATORIO)

Compared with OPERA, similar covariates were associated with a higher risk of SIs in patients with PPMS in the univariate analysis (Supplemental Figure 6), including being located in the United States (2.40 [95% CI: 1.30–4.43]), having overweight or obesity (BMI > 25 kg/m^2^, 2.12 [95% CI: 1.39–3.22]), IgM < LLN (2.04 [95% CI: 1.46–2.85]), and the presence of ⩾2 comorbidities (2.60 [95% CI: 1.53–4.40]), of which renal and urinary, depression, cardiovascular comorbidities, and previous infections were the most significant in a descending order (Supplemental Figure 6). However, requiring assistance to walk or being nonambulatory was associated with the greatest risk (3.96 [95% CI: 1.96–8.00]). Lymphopenia was not associated with an increased risk of SIs (1.28 [95% CI: 0.48–3.46]).

After adjusting for multiple covariates (Figure 7(b)), having an EDSS score ⩾6.0 remained the highest risk for SIs, associated with a 4-fold increased risk (4.31 [95% CI: 2.09–8.87]). Abnormal IgM levels (1.89 [95% CI: 1.29–2.79]), the presence of ⩾2 comorbidities (1.80 [95% CI: 1.06–3.08]), and having overweight or obesity (1.46 [95% CI: 1.00–2.13]) also retained an association with an increased risk of SIs; it should be noted that among 234 patients with IgM < LLN, 6 (2.6%) patients experienced 29 out of 78 (37.2%) SIs, suggesting a potentially skewed risk association.

The results of both univariate and multivariate models for all infections were overall consistent with the results observed for SIs (Figure 7(b) and Supplemental Figure 6). In the multivariate model, having a higher comorbidity burden (⩾2 comorbidities, 1.63 [95% CI: 1.36–1.95]; one comorbidity, 1.42 [95% CI: 1.20–1.67]), having underweight (1.60 [95% CI: 1.13–2.28]), being located in the United States (1.33 [95% CI: 1.08–1.64]), and having greater disability (EDSS score ⩾6.0, 1.31 [95% CI: 1.08–1.59]) were independent risk factors. Similar to the observation in the RMS population, male patients with PPMS also exhibited a reduced risk of infections (0.73 [95% CI: 0.62–0.86]) compared with female patients with PPMS, and time on OCR (0.96 [95% CI: 0.94–0.97]) was not associated with an increased risk of infections.

## Discussion

The present analysis provides a comprehensive assessment of the incidence, clinical characteristics, and outcomes of SIs in a large, heterogeneous MS population (*n* = 6155) treated continuously with OCR for up to 14 years across 13 clinical trials. It also includes the most complete dataset for assessment of potential risk factors in 2092 PwMS with a follow-up of up to 12 years.

UTIs, LRTIs, gastrointestinal infections, and skin infections were the most commonly reported types of SIs, similar to evidence for anti-CD20 drugs^23^ and reports from real-world studies.^1,3–6,22^ Nearly 90% of all SIs resolved without sequelae, and <0.5% of all patients discontinued treatment due to SIs. When COVID-19 cases were excluded, only 8 infection-related fatalities (0.03 per 100 PY [95% CI: 0.01–0.06]) were reported in 6155 OCR-treated patients, well below fatal infection rates reported in real-world MS studies (0.1 per 100 PY),^3^ suggesting a manageable infection risk profile.

Rates of SIs remained stable and infrequent, with only minor fluctuations observed over time, including in the subset of patients (*n* = 2092) from the pivotal phase III trials. These results are discordant with a recent large real-world study of patients treated with anti-CD20 agents (rituximab and OCR) over a mean time of 2.6 years^24^; total time on rituximab/OCR modestly increased annual risk for infections resulting in hospitalization, intravenous antibiotics, or extended dosing antibiotics (odds ratio [OR]: 1.33 [95% CI: 1.17–1.51]). However, it should be noted that this study included PwMS and neuromyelitis optica spectrum disorder, and was conducted on complete case analysis, excluding many patients with incomplete or missing data. The authors were also unable to account for some risk factors such as BMI and comorbidities. As shown in our univariate analysis, the presence of cardiovascular disease, depression, previous infections, and renal and urinary comorbidities was generally associated with an increased risk of infections and SIs. While depression may seem a biologically implausible risk factor, the same association was previously observed in a large study investigating multiple Danish registries.^25^ In the present study, patients with PPMS who have overweight/obesity also had an elevated risk for SIs, notably sepsis and skin infections, while patients with PPMS who have underweight had a higher risk of all infections. A U-shaped increased infection rate has been suggested in both adults who have underweight and obesity.^26^ These results have clear implications for the management of OCR-treated PwMS, as many of these comorbidities can to some extent be modified, which may help mitigate safety risks.

In our analysis, patients with RMS experiencing clinical relapses during the study had a 2-fold increased risk of SIs. Patients with PPMS requiring bilateral walking assistance or a wheelchair had an over 4-fold increased risk of SIs, in agreement with an 8.6-fold increased risk of SIs in wheelchair-bound patients previously shown by Vollmer et al.^24^ The clinical benefit of an earlier intervention with OCR, demonstrated on outcomes such as relapses and in particular disability,^27,28^ may therefore result in a lowering of the risk of SIs.

The associations of SIs with lymphopenia and neutropenia have been previously characterized^10^; the current analysis confirms that these laboratory abnormalities occur infrequently and are not associated with increased risk of SIs in OCR-treated patients. The potential risk posed by low IgG levels remains one of the greatest concerns among neurologists treating patients with anti-CD20 drugs such as OCR, despite other non-anti-CD20 DMTs such as natalizumab, fingolimod, and teriflunomide also potentially leading to reduced IgG levels.^23,29–31^ Our analysis showed that while IgG (and IgM) levels decrease over a period of 12 years, for more than 8 out of 10 patients, IgG levels remain normal and in half of the cases, periods of low IgG are transient and return to normal after approximately three infusion cycles. Importantly, discontinuation due to low Ig is uncommon, as is the need for IVIg replacement therapy. Other anti-CD20s with a shorter follow-up also have a limited impact on patient IgG levels, with few patients discontinuing due to low IgG levels.^23,32^ It also demonstrated that while the risk for SIs in patients experiencing hypogammaglobulinemia is seen in the univariate analyses, when accounting for multiple risk factors, decreased IgG levels were not significantly associated with SIs. In fact, rates of SIs associated with low IgG remained stable over a period of up to 12 years, with <2% of patients experiencing SIs during periods of IgG < LLN; the type, severity, duration, and outcome of these SIs are consistent with SIs observed in the overall OCR-treated population. In Vollmer et al.,^24^ patients with IgG <500 mg/dL showed increased odds of SIs (adjusted OR: 3.15); however, missing data were common for IgG values as these were not regularly collected. It is possible that Ig levels may have been more frequently measured in patients who were already presenting with infections, in contrast with our analyses, where IgG values were measured in all patients at regular intervals. An association between IgM < LLN and SIs in patients with PPMS was observed, but this was not evident in patients with RMS. This might be a chance finding due to the skewness of the data, given that 37% of SIs occurring during periods of IgM < LLN were observed in only 2.6% of patients. Vollmer et al.^24^ showed no association between low IgM and infections.

The current analysis also highlights important information on infections of special interest in MS. For example, no clinical or laboratory reactivation of HBV was reported in 175 patients with a positive HBcAb titer at baseline. Only around 7% of these patients were on prophylactic antiviral treatment, suggesting that the risk of reactivation in PwMS might be low; outside of clinical trial settings, one case of HBV reactivation has been published.^33^ While vaccination remains the best strategy to prevent HBV infection, our findings are consistent with recent recommendations that regular monitoring of HBcAb+ patients via HBV DNA quantification (performed every 6 months in OCR clinical trials) can be used as a possible risk mitigation strategy for patients at risk of reactivation, beyond other more common approaches such as the initiation of prophylaxis with antiviral drugs.^34,35^ Opportunistic infections of general concern for clinicians treating PwMS^36–40^ were not observed in this analysis, and serious herpes infections were infrequently reported. Reports on COVID-19 cases in patients treated with OCR have been previously published,^41,42^ and at the time of writing, detailed analyses including both clinical and immunological (B-cell and T-cell levels) outcomes, are underway.

The current analysis has some limitations. This is a pooled, *post-hoc* analysis based on patients enrolled into clinical trials, limiting generalizability to real-world settings. Still, the cohort had a very heterogeneous composition, including patients across the entire MS disease spectrum, with age above the typical upper limit in clinical trials (>65 years), long DMT histories, and a higher number of comorbidities. Attrition bias may also be a potential limitation as patients who discontinued earlier from the OPERA and ORATORIO studies were more likely to have higher disability levels, but interestingly, a similar comorbidity burden as those who completed the studies, at the time of randomization and discontinuation (Supplemental Table 10). Finally, although the study included long-term data, it should be noted that patient numbers beyond Year 11 of treatment are limited. This analysis has several strengths; it is the largest cohort of OCR-treated patients, across multiple countries with diverse standards of MS care, and it is also unique for its prospective nature and the long follow-up period. This is also the most detailed analysis to date investigating risk factors for infections and SIs with an anti-CD20 treatment.

## Conclusion

In conclusion, the main findings of this integrated pooled analysis suggest that continuous long-term treatment with OCR is associated with a manageable infection risk profile, with rates of SIs remaining low and stable, with infections rarely leading to unfavorable clinical outcomes, and with most patients continuing treatment with OCR. Longer-term follow-up and post-marketing studies will continue to monitor the infection risk profile of long-term treatment with OCR.

## Supplemental Material

sj-docx-1-tan-10.1177_17562864241277736 – Supplemental material for Long-term analysis of infections and associated risk factors in patients with multiple sclerosis treated with ocrelizumab: pooled analysis of 13 interventional clinical trials Supplemental material, sj-docx-1-tan-10.1177_17562864241277736 for Long-term analysis of infections and associated risk factors in patients with multiple sclerosis treated with ocrelizumab: pooled analysis of 13 interventional clinical trials by Tobias Derfuss, Robert Bermel, Chien-Ju Lin, Stephen L. Hauser, Ludwig Kappos, Timothy Vollmer, Giancarlo Comi, Gavin Giovannoni, Hans-Peter Hartung, Martin S. Weber, Jianmei Wang, Nikki Jessop, Cathy Chognot, Licinio Craveiro and Amit Bar-Or in Therapeutic Advances in Neurological Disorders

sj-docx-2-tan-10.1177_17562864241277736 – Supplemental material for Long-term analysis of infections and associated risk factors in patients with multiple sclerosis treated with ocrelizumab: pooled analysis of 13 interventional clinical trials Supplemental material, sj-docx-2-tan-10.1177_17562864241277736 for Long-term analysis of infections and associated risk factors in patients with multiple sclerosis treated with ocrelizumab: pooled analysis of 13 interventional clinical trials by Tobias Derfuss, Robert Bermel, Chien-Ju Lin, Stephen L. Hauser, Ludwig Kappos, Timothy Vollmer, Giancarlo Comi, Gavin Giovannoni, Hans-Peter Hartung, Martin S. Weber, Jianmei Wang, Nikki Jessop, Cathy Chognot, Licinio Craveiro and Amit Bar-Or in Therapeutic Advances in Neurological Disorders

## Declarations

**Ethics approval and consent to participate:** Protocols were approved by the relevant institutional review boards/ethics committees, and all patients provided written informed consent. Our analysis of clinical trial data did not require one singular ethical board approval because all analyzed data were derived from multiple separate trials that were all reviewed and approved by the local ethical boards. Approved studies included: Phase II (NCT00676715), OPERA I (NCT01247324), OPERA II (NCT01412333), ORATORIO (NCT01194570), VELOCE (NCT02545868), CHORDS (NCT02637856), CASTING (NCT02861014), OBOE (NCT02688985), ENSEMBLE (NCT03085810), CONSONANCE (NCT03523858), LIBERTO (NCT03599245), CHIMES (NCT04377555), and OLERO (NCT05269004).

**Consent for publication:** Not applicable.

**Competing interests:** The authors declared the following potential conflicts of interest with respect to the research, authorship, and/or publication of this article: T.D. has served on scientific advisory boards, steering committees, and data safety monitoring boards for Actelion, Biogen, Celgene, Genzyme, GeNeuro, Merck, Mitsubishi Pharma, Novartis, Roche, Octapharma, and MedDay; has received travel and/or speaker honoraria from Biogen, Genzyme, Merck, Novartis, Roche, and Merck-Serono; and has received research support from Biogen, Novartis, the Swiss MS Society, and the Swiss National Foundation. R.B. has served as a consultant for Biogen, EMD Serono, Genzyme, Genentech, Inc., Novartis, and Viela Bio. He receives research support from Biogen, Genentech, Inc., and Novartis; and shares rights to intellectual property underlying the Multiple Sclerosis Performance Test, currently licensed to Qr8 Health and Biogen. C.-J.L. is an employee of F. Hoffmann-La Roche Ltd. S.L.H. is on the editorial board of *Therapeutic Advances in Neurological Disorders*, serves on scientific advisory boards for Accure, Alector, and Annexon; previously consulted for BD, Moderna, NGM Bio, and Pheno Therapeutics; previously served on the Board of Directors for Neurona; and has received nonfinancial support (travel reimbursement and writing assistance for anti-CD20-related meetings and presentations) from Roche and Novartis. L.K.’s institution (University Hospital Basel) has received the following exclusively for research support: steering committee, advisory board, and consultancy fees (Actelion, Bayer HealthCare, Biogen, Bristol Myers Squibb, Genzyme, Janssen, Japan Tobacco, Merck, Novartis, Roche, Sanofi, Santhera, Shionogi, and TG Therapeutics); speaker fees (Bayer HealthCare, Biogen, Merck, Novartis, Roche, and Sanofi); support of educational activities (Allergan, Bayer HealthCare, Biogen, CSL Behring, Desitin, Genzyme, Merck, Novartis, Roche, Pfizer, Sanofi, Shire, and Teva); license fees for Neurostatus products; and grants (Bayer HealthCare, Biogen, European Union, InnoSwiss, Merck, Novartis, Roche, the Swiss MS Society, and the Swiss National Research Foundation). T.V. has received compensation for consultancy from Biogen, Genentech/Roche, and Novartis and has received research support from Rocky Mountain Multiple Sclerosis Center, Celgene, Biogen, Anokion, Genentech, Inc., F. Hoffmann-La Roche Ltd, GW Pharma, and TG Therapeutics. G.C. has received consultancy and speaking fees from Novartis, Sanofi-Genzyme, Genzyme, Merck KGgA, Merck-Serono SpA, Celgene, F. Hoffmann-La Roche Ltd, Almirall SpA, and Janssen. G.G. has received personal compensation for serving as a consultant for F. Hoffmann-La Roche Ltd, AbbVie, Actelion, Atara Biotherapeutics, Biogen, Celgene, Sanofi-Genzyme, Genentech, Inc., GlaxoSmithKline, Merck-Serono, Novartis, and Teva; has received personal compensation from Elsevier for serving as an editor on *MSARD*s; and has received financial support for research activities from F. Hoffmann-La Roche Ltd, Biogen, Merck, Merck-Serono, Novartis, Sanofi-Genzyme, and Takeda. H.-P.H. has received honoraria for consultancy, serving on steering committees, and speaking at scientific symposia with approval by the Rector of Heinrich-Heine University Düsseldorf from Bayer, Biogen, F. Hoffmann-La Roche Ltd, GeNeuro SA, Genzyme, MedImmune, Merck-Serono, Novartis, Octapharma, Sanofi-Genzyme, Teva, TG Therapeutics, and Viela Bio. H.-P.H. is also on the Editorial Board Member of *Therapeutic Advances in Neurological Disorders*; therefore, the peer-reviewer process for this manuscript was managed by alternative members of the Board and the submitting Editor was not involved in the decision-making process. M.S.W. receives research support from the Deutsche Forschungsgemeinschaft (DFG; WE 3547/5-1), Novartis, Teva, Biogen Idec., Roche, Merck, and the ProFutura Program of the Universitätsmedizin Göttingen; is serving as an editor for *PLoS One*; and received travel funding and/or speaker honoraria from Biogen Idec, Merck-Serono, Novartis, Roche, Teva, Bayer, and Genzyme. J.W. is an employee of F. Hoffmann-La Roche Ltd. N.J. is an employee of F. Hoffmann-La Roche Ltd. C.C. is an employee of and a shareholder in F. Hoffmann-La Roche Ltd. L.C. is an employee of and a shareholder in F. Hoffmann-La Roche Ltd. A.B.-O. has received consultancy fees from Actelion, Atara Biotherapeutics, Biogen Idec, Celgene/Receptos, Genentech/Roche, MAPI, MedImmune, Merck/EMD Serono, Novartis, Sanofi-Genzyme, GlaxoSmithKline, and Brainstorm; carried out contracted research for Genentech, Inc., and Biogen; and receives a salary from the University of Pennsylvania, Perelman School of Medicine.

**Availability of data and materials:** Qualified researchers may request access to individual patient level data through the clinical study data request platform (https://vivli.org/). Further details on Roche’s criteria for eligible studies are available here: https://vivli.org/members/ourmembers/. For further details on Roche’s Global Policy on the Sharing of Clinical Information and how to request access to related clinical study documents, see here: https://www.roche.com/research_and_development/who_we_are_how_we_work/clinical_trials/our_commitment_to_data_sharing.htm.
