# Supplementary material for: Long-term analysis of infections and associated risk factors in patients with multiple sclerosis treated with ocrelizumab: pooled analysis of 13 interventional clinical trials
Source: Ther Adv Neurol Disord. 2024 Oct 8;17:17562864241277736. doi: 10.1177/17562864241277736 (PMC11470513; doi:10.1177/17562864241277736)
Supplement: sj-docx-1-tan-10.1177_17562864241277736 – Supplemental material for Long-term analysis of infections and associated risk factors in patients with multiple sclerosis treated with ocrelizumab: pooled analysis of 13 interventional clinical trials [file sj-docx-1-tan-10.1177_17562864241277736.docx]

# Supplementary Figures

**
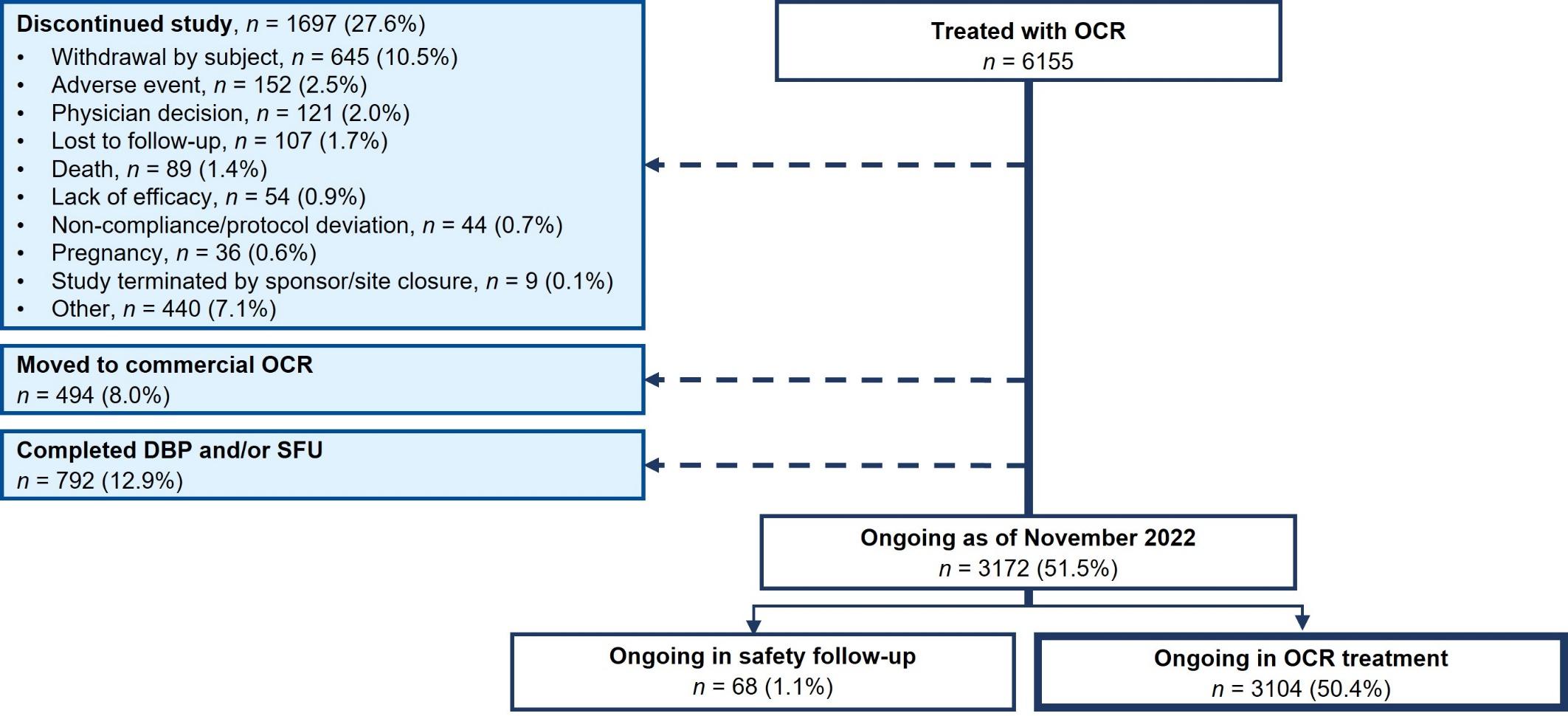
**

**Figure S1.** CONSORT flow diagram.

Data cut-off: November 2022.

DBP, double-blind period; OCR, ocrelizumab; SFU safety follow-up.


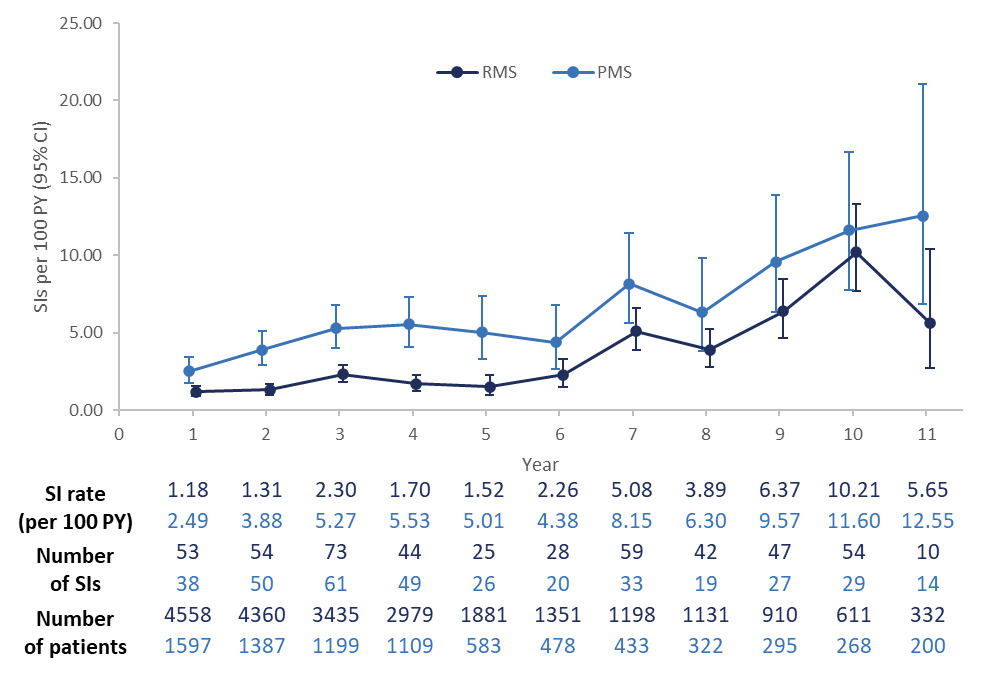


**Figure S2.** Yearly rates of SIs in RMS and PMS populations with COVID-19 events included.

Pooled data on yearly rates of SIs from the 13 OCR clinical trials are presented. Patients with longer exposure (≥6 years) are from the extension periods of the phase II and phase III studies, including those originally randomized to the comparators interferon β-1a (OPERA) or placebo (ORATORIO) who switched to open-label OCR treatment. Patients with exposure beyond 11 years (up to 14 years) are not represented in the graph. CCOD, November 2022.

CCOD, clinical cut-off date; CI, confidence interval; COVID-19, coronavirus disease 2019; OCR, ocrelizumab; PMS, progressive multiple sclerosis; PY, patient years; RMS, relapsing multiple sclerosis; SI, serious infection.


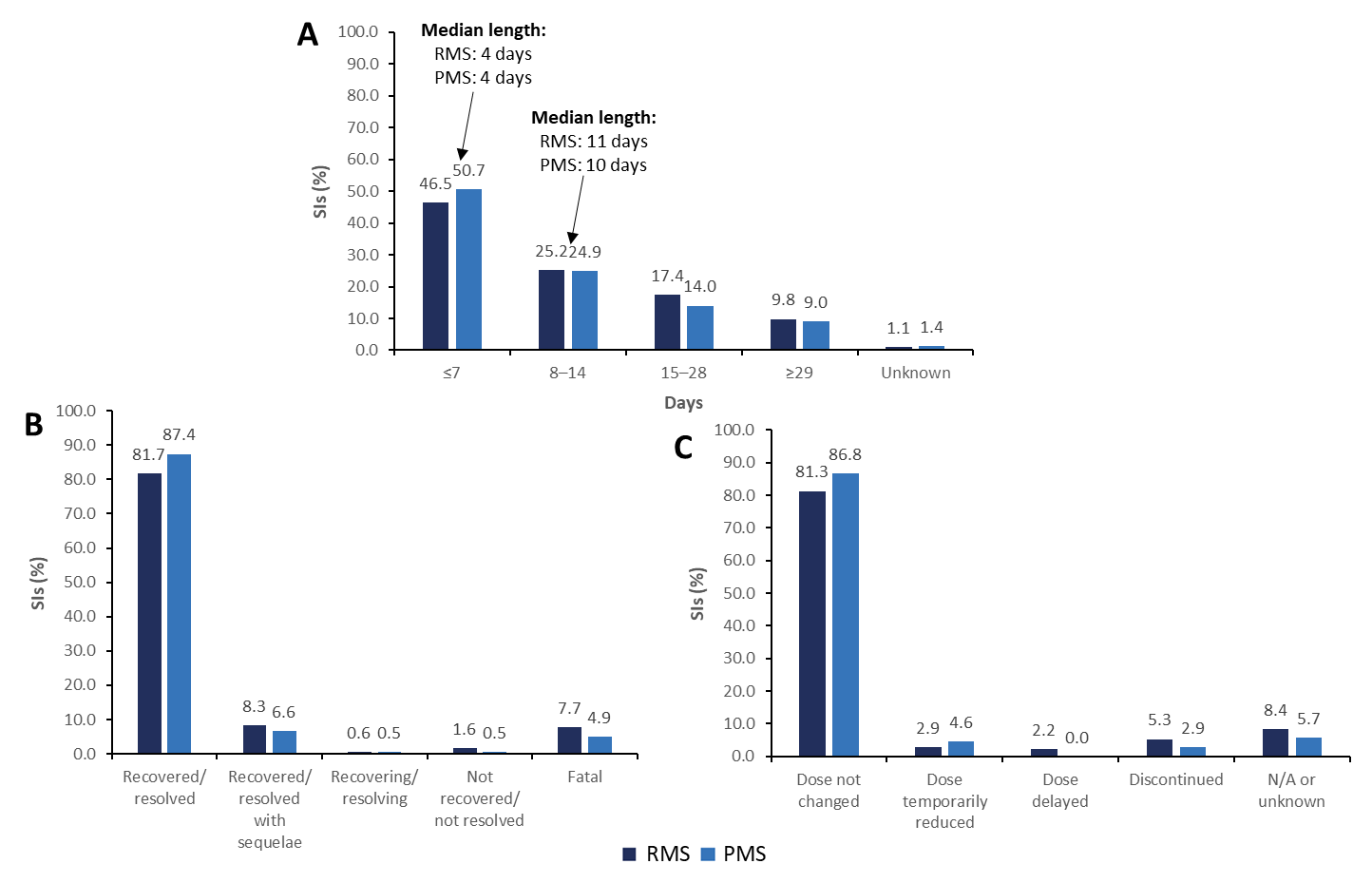
**Figure S3.** Outcomes and action taken with OCR following SIs, including COVID-19.

(A) Length of hospitalization for SIs requiring admission to hospital (*n* = 794/858) in patients with RMS and PMS. (B) Outcomes of SIs in patients with RMS and PMS; fatal cases include COVID-19 (*n* = 48), pneumonia (*n* = 2), sepsis (*n* = 2), encephalitis (*n* = 1), enterococcal infection (*n* = 1), pneumonia aspiration (*n* = 1), and urosepsis (*n* = 1). (C) SIs grouped according to action taken with OCR. CCOD, November 2022.

CCOD, clinical cut-off date; COVID-19, coronavirus disease 2019; N/A, not applicable; OCR, ocrelizumab; PMS, progressive multiple sclerosis; RMS, relapsing multiple sclerosis; SI, serious infection.


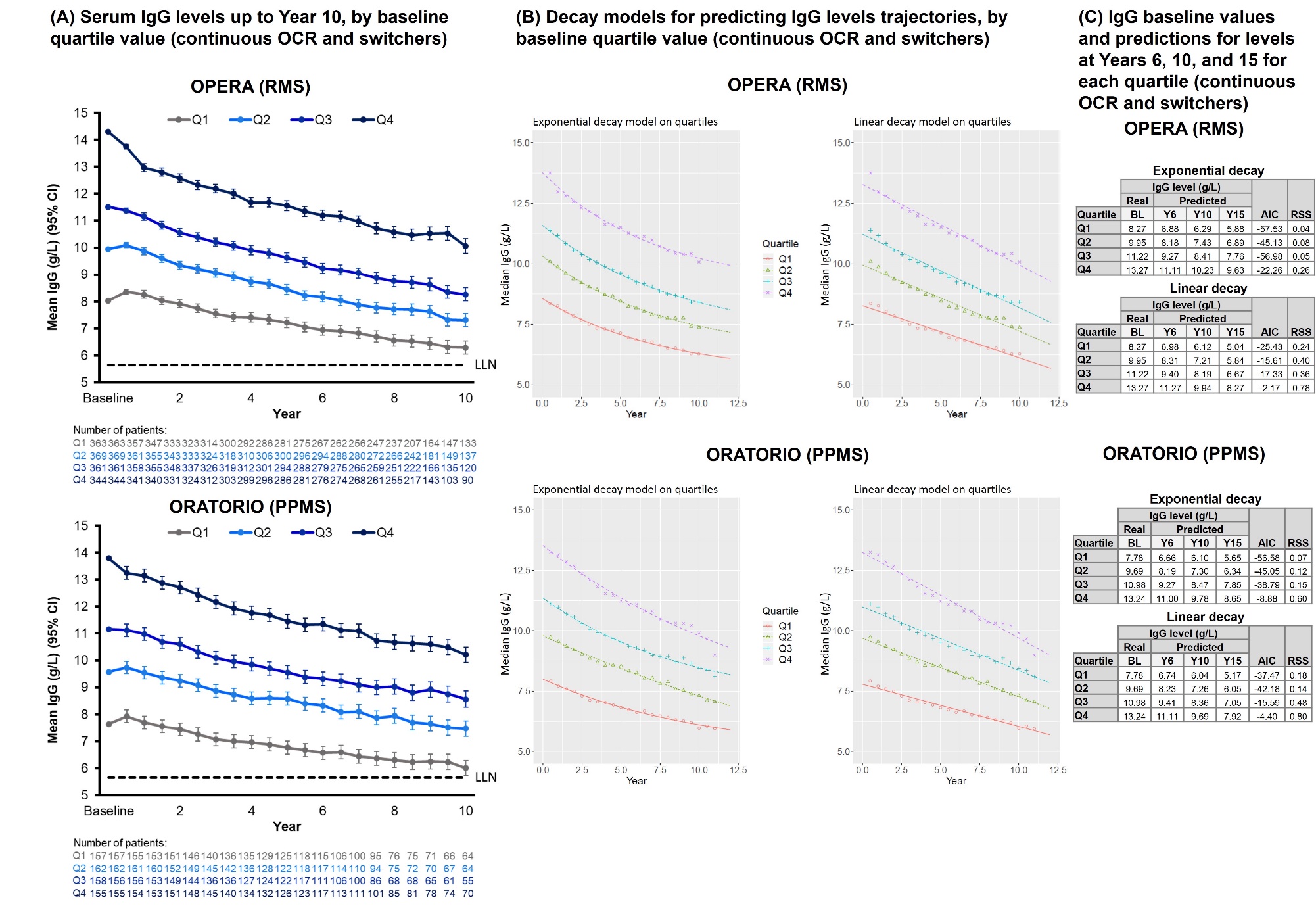


**
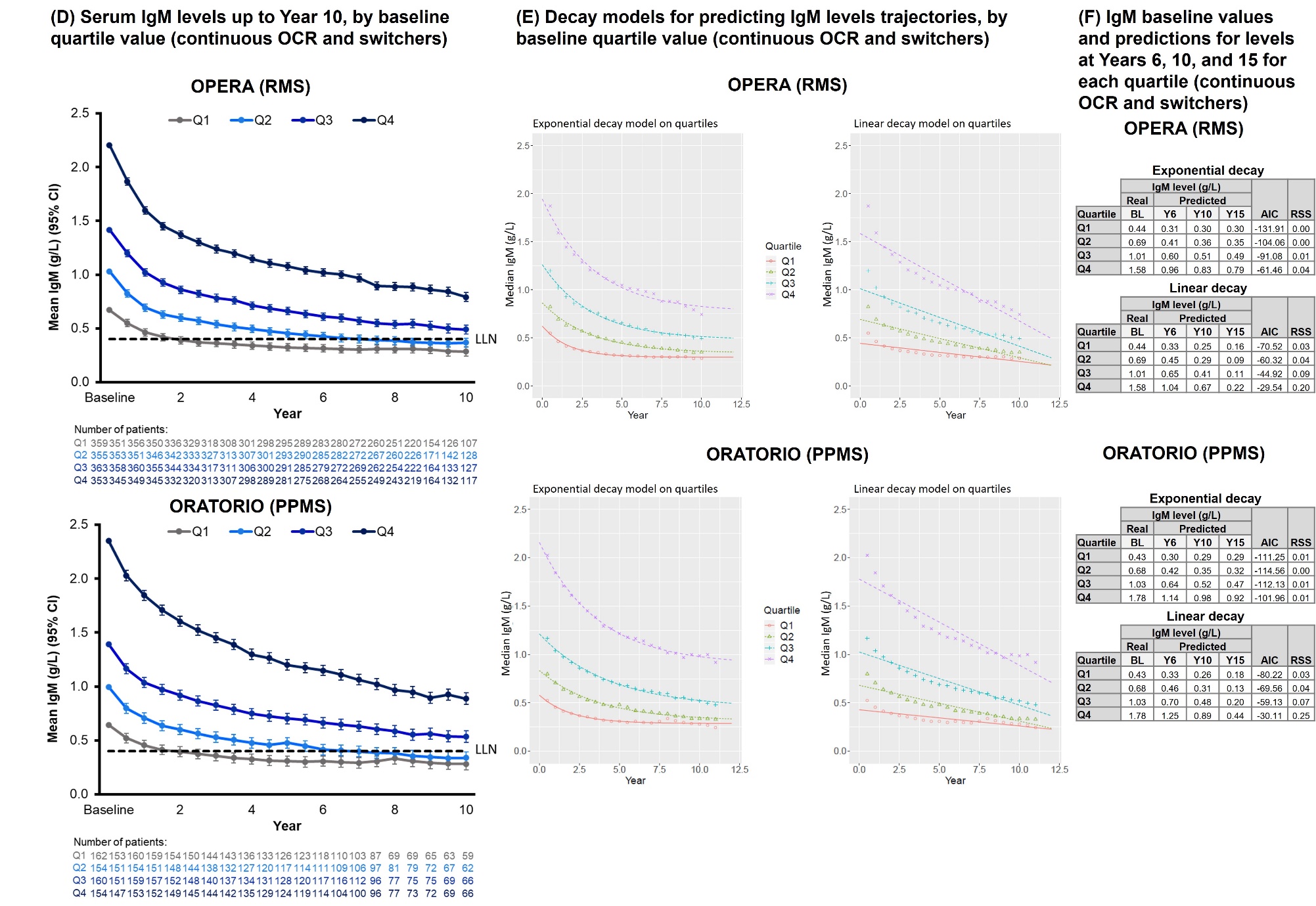
**

**Figure S4.** Yearly IgG and IgM levels by baseline quartiles.

**(A)** IgG trajectories by quartile according to BL levels in OPERA (top) and ORATORIO (bottom); quartile thresholds are displayed. IgG LLN = 5.65 g/L. **(B)** Decay models for estimating IgG level trajectories up to 12 years. For each cohort (OPERA and ORATORIO) and each quartile, an exponential and a linear model were fit; exponential decay model: y=y_0_(1-λ)^t^, where y_0_ is the y at BL, t is the time, λ is the decay factor. Linear model: y=β_0_+β_1_t... **(C)** Predicted IgG levels at Years 6 and 10 (within the data range) and Year 15 (outside the data range) for each model. Lower AIC values indicate a better-fit model. AIC values were lower for the exponential models compared with linear models. In addition, the exponential models had a smaller RSS, indicating that the exponential models had a better fit. **(D)** IgM trajectories by quartile according to BL levels in OPERA (top) and ORATORIO (bottom); quartile thresholds are displayed. IgM LLN = 0.4 g/L. **(E)** Decay models for estimating IgM level trajectories up to 12 years. For each cohort (OPERA and ORATORIO) and each quartile, an exponential and a linear model were fit; exponential decay model: y=y_0_(1-λ)^t^, where y_0_ is the y at BL, t is the time, λ is the decay factor. Linear model: y=β_0_+β_1_t… **(F)** Predicted IgM levels at Years 6 and 10 (within the data range) and Year 15 (outside the data range) for each model. Lower AIC values indicate a better-fit model. AIC values were lower for the exponential models compared with linear models. In addition, the exponential models had smaller residual sum of squares, which indicates that the exponential models had a better fit.

AIC, Akaike information criterion; BL, baseline; CI, confidence interval; Ig, immunoglobulin; LLN, lower limit of normal; OCR, ocrelizumab; PPMS, primary progressive multiple sclerosis; Q, quartile; RMS, relapsing multiple sclerosis; RSS, residual sum of squares; Y, year.


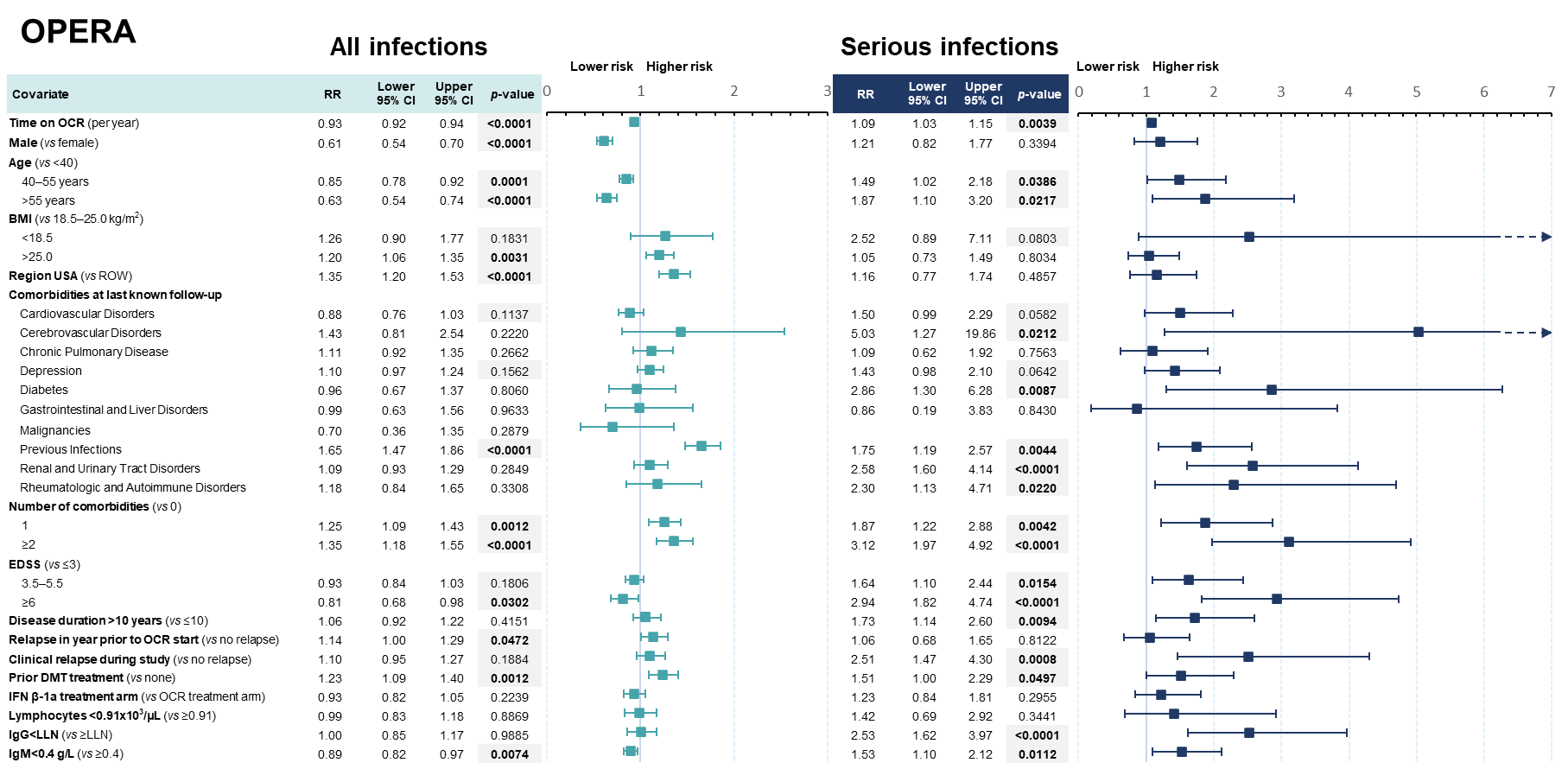


**Figure S5.** Univariate model for risk of infections and serious infections in OPERA (RMS).

For all the lab assessments, the median of the infusion cycle is presented. A hypothetical infusion cycle is a 180-day period from the date of the last infusion. EDSS at unscheduled visits and within up to 30 days after the onset of a protocol-defined relapse to confirm progression were excluded. Time-varying covariates: Age, comorbidity, EDSS, lymphocytes, neutrophils, IgG, IgG quartiles, IgM, year. Shaded boxes indicate *p* < 0.2, the cut-off value that was used to determine the risk factors used for the multivariate model. *p*-values below 0.05 are marked in bold.

BMI, body mass index; CI, confidence interval; DMT, disease-modifying therapy; EDSS, Expanded Disability Status Scale; IFN, interferon; Ig, immunoglobulin; LLN, lower limit of normal; OCR, ocrelizumab;
RMS, relapsing multiple sclerosis; ROW, rest of the world; RR, rate ratio.


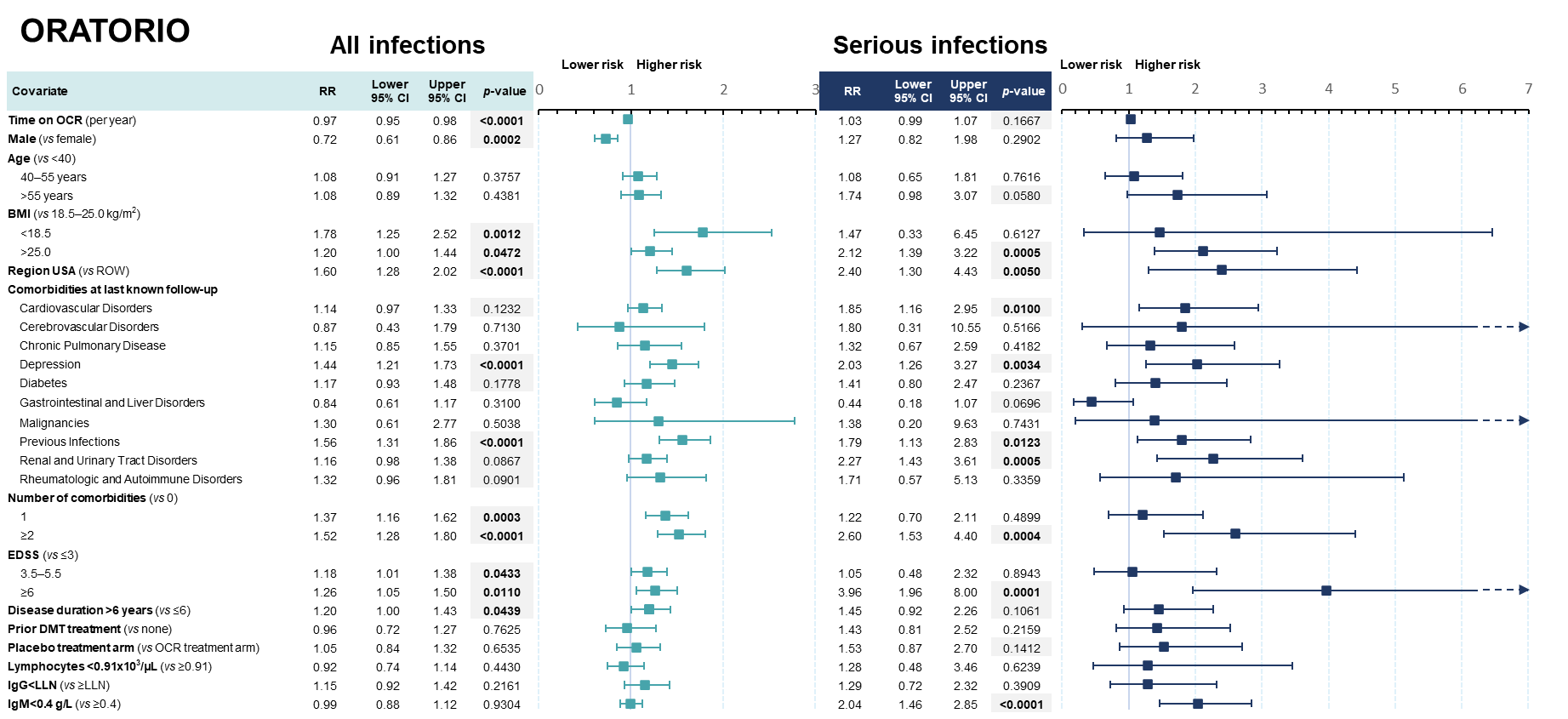


**Figure S6.** Univariate model for risk of infections and serious infections in ORATORIO (PPMS).

Four placebo patients who received OCR before OLE were removed from the analysis. For all the lab assessments, the median of the infusion cycle is presented. A hypothetical infusion cycle is a 180-day period from the date of the last infusion. EDSS at unscheduled visits and within up to 30 days after the onset of a protocol-defined relapse to confirm progression were excluded. Time-varying covariates: Age, comorbidity, EDSS, lymphocytes, neutrophils, IgG, IgG quartiles, IgM, year. Shaded boxes indicated *p* < 0.2, the cut-off value that was used to determine the risk factors used for the multivariate model. *p*-values below 0.05 are marked in bold.

BMI, body mass index; CI, confidence interval; DMT, disease-modifying therapy; EDSS, Expanded Disability Status Scale; Ig, immunoglobulin; LLN, lower limit of normal; OCR, ocrelizumab; OLE, open-label extension; PPMS, primary progressive multiple sclerosis; ROW, rest of the world; RR, rate ratio.

# Supplementary Figure References

1. Kappos L, Li D, Calabresi PA, *et al.* Ocrelizumab in relapsing-remitting multiple sclerosis: a phase 2, randomised, placebo-controlled, multicentre trial. *Lancet* *(London, England)* 2011; 378: 1779–1787.

2. Hauser SL, Bar-Or A, Comi G, *et al.* Ocrelizumab versus interferon beta-1a in relapsing multiple sclerosis. *N Engl J Med* 2017; 376: 221–234.

3. Montalban X, Hauser SL, Kappos L, *et al.* Ocrelizumab versus placebo in primary progressive multiple sclerosis. *N Engl J Med* 2017; 376: 209–220.
