# Supplementary material for: Long-term analysis of infections and associated risk factors in patients with multiple sclerosis treated with ocrelizumab: pooled analysis of 13 interventional clinical trials
Source: Ther Adv Neurol Disord. 2024 Oct 8;17:17562864241277736. doi: 10.1177/17562864241277736 (PMC11470513; doi:10.1177/17562864241277736)
Supplement: sj-docx-2-tan-10.1177_17562864241277736 – Supplemental material for Long-term analysis of infections and associated risk factors in patients with multiple sclerosis treated with ocrelizumab: pooled analysis of 13 interventional clinical trials [file sj-docx-2-tan-10.1177_17562864241277736.docx]

# Supplementary Tables

# Table S1. Overview of study designs.

| **Study name**  **(NCT number)** | **Population (start date)** | **Naive/**  **pretreated (%)^a^** | **Design**  **(phase)** | **Treatment** | **Number of patients** | **Overall PY**  **(Nov 2022)** | **Duration of**  **main study** | **Entering**  **OLE?^b^** | **Objective**  **of study** | **Primary**  **endpoint** | **Current status** |
| --- | --- | --- | --- | --- | --- | --- | --- | --- | --- | --- | --- |
| **Phase II**  **(NCT00676715)** | RRMS  (2008) | Naive and pretreated (41.3%) | Controlled  (phase II) | OCR 2000 mg every 6M^c,d^ | 55 | 1458 | 96 weeks | Yes | Evaluate the efficacy and safety of 2 dose regimens of OCR | Total number of T1 Gd-enhancing lesions observed on brain MRI scans at Weeks 12, 16, 20, and 24 | Active, not recruiting |
|  |  |  |  | OCR 600 mg every 6M^e^ | 55 |  |  |  |  |  |  |
|  |  |  |  | Placebo every 6M^f^ | 54 |  |  |  |  |  |  |
|  |  |  |  | IFN β-1a 30 μg once weekly^f^ | 54 |  |  |  |  |  |  |
| **OPERA I**  **(NCT01247324)** | RMS  (2011) | Naive and pretreated (27.4%) | Controlled  (phase III) | OCR 600 mg every 6M^e^ | 410 | 5537 | 96 weeks | Yes | Evaluate the efficacy and safety of OCR in comparison to IFN β-1a | ARR IFN β-1a at Week 96 | Completed (2022) |
|  |  |  |  | IFN β-1a 44 μg three times weekly | 411 |  |  |  |  |  |  |
| **OPERA II**  **(NCT01412333)** | RMS  (2011) | Naive and pretreated (25.9%) | Controlled  (phase III) | OCR 600 mg every 6M^e^ | 417 | 5262 | 96 weeks | Yes | Evaluate the efficacy and safety of OCR in comparison to IFN β-1a | ARR at Week 96 | Completed  (2022) |
|  |  |  |  | IFN β-1a 44 μg three times weekly | 418 |  |  |  |  |  |  |
| **ORATORIO**  **(NCT01194570)** | PPMS  (2011) | Naive and pretreated (11.6%) | Controlled  (phase III) | OCR 600 mg every 6M^g^ | 488 | 4669 | 120 weeks | Yes | Evaluate the efficacy and safety of OCR in comparison to placebo | Percentage of patients with CDP sustained for at least 12 weeks | Completed (2022) |
|  |  |  |  | Placebo every 6M | 244 |  |  |  |  |  |  |
| **VELOCE**  **(NCT02545868)** | RMS  (2015) | Naive and pretreated (41.2%) | Controlled  (phase IIIb) | OCR 600 mg every 6M^e^ | 68 | 472 | 24 weeks | Yes | Evaluate the immune response to vaccines after administration of OCR | Percentage of patients with a positive response 8 weeks post-tetanus vaccine | Completed  (2021) |
|  |  |  |  | IFN β-1a or no DMT | 34 |  |  |  |  |  |  |
| **CHORDS**  **(NCT02637856)** | RRMS  (2016) | Pretreated (100.0%) | Single arm  (phase IIIb) | OCR 600 mg every 6M^e^ | 611 | 1116 | 96 weeks | No^h^ | Evaluate the efficacy and safety of OCR in patients with a suboptimal response to a DMT in the USA/Canada | Percentage of patients free from any protocol-defined event during 96-week period, including occurrence of at least one of the following: a protocol-defined relapse, a T1 Gd-enhancing lesion, a new and/or enlarging T2 lesion, or CDP at 24 weeks | Completed  (2019) |
| **CASTING**  **(NCT02861014)** | RRMS  (2016) | Pretreated (100.0%) | Single arm  (phase IIIb) | OCR 600 mg every 6M^e^ | 681 | 516 | 96 weeks | Yes | Evaluate the efficacy and safety of OCR in patients with a suboptimal response to a DMT in Europe | Percentage of patients with NEDA (as per protocol-defined events) during 96-week period | Completed  (2020) |
| **OBOE**  **(NCT02688985)** | RMS  (2016) | Naive and pretreated (37.5%) | Single arm  (phase IIIb) | OCR 600 mg every 6M^e^ | 88 | 492 | 52 weeks | Yes | Explore the mechanism of action of OCR and B-cell biology | Change in NfL levels in the CSF from baseline to post-treatment with OCR; changes in the numbers of CD19+ B cells and CD3+ T cells in the CSF from baseline to post-treatment with OCR | Completed  (2023) |
|  | PPMS  (2016) |  |  | OCR 600 mg every 6M^g^ | 16 |  |  |  |  |  |  |
| **ENSEMBLE**  **(NCT03085810)** | Early-stage  RRMS  (2017) | Naive (100.0%) | Single arm  (phase IIIb) | OCR 600 mg every 6M^e^ | 1233^i^ | 2181 | 192 weeks | Yes | Evaluate the efficacy and safety of OCR | A set of endpoints addressing different facets of MS: clinical assessments conducted every 24 weeks; MRI conducted at Weeks 8 (rebaselining), 24, 48, 96, 144, and 192 | Completed  (2023) |
| **LIBERTO**  **(NCT03599245)** | MS  (2018) | Pretreated (100.0%) | Single arm  (phase IIIb) | OCR 600 mg every 6M^e^ | 1500^i^ | 3949 | 144 weeks | Yes | Long-term extension study to CASTING and ENSEMBLE to evaluate the efficacy and safety of OCR | Effectiveness of OCR in patients with MS assessed by progression of disease up to 2 years | Active, not recruiting |
| **CONSONANCE**  **(NCT03523858)** | PMS (PPMS  and SPMS)  (2018) | Naive and pretreated (65.1%) | Single arm  (phase IIIb) | OCR 600 mg every 6M^j^ | 922 | 2393 | 192 weeks | Yes | Open-label study to evaluate the effectiveness and safety of OCR in patients with PMS | Percentage of patients with NEP and NEPAD from baseline to Week 96, Week 96 to Week 192, and baseline to Week 192 | Active, not recruiting |
| **OLERO**  **(NCT05269004)** | RMS and  PPMS  (2022) | Pretreated (100.0%) | Single arm  (phase IIIb) | OCR 600 mg every 6M^j^ | ~1300 | 0 | 192 weeks | Yes | Open-label study to evaluate the effectiveness and safety of OCR in patients with MS who were previously enrolled in phase III studies | Incidence and severity of adverse events, with severity determined according to the NCI CTCAE from baseline to Week 192 | Recruiting |
| **CHIMES**  **(NCT04377555)** | RMS  (2022) | Naive or initiating 1st or 2nd switch from treatment with certain DMTs | Single arm  (phase IV) | OCR 600 mg every 6M^j^ | 182 | 225 | 48 weeks (optional 168 weeks extension) | No | Open-label study to assess disease activity biomarkers of neuronal damage in minority patients with RMS | Proportion of participants free of any protocol-defined events (relapse, progression, a T1 Gd-enhancing lesion, a new and/or enlarging T2 lesion) from baseline to Week 48 | Active, not recruiting |

^a^Percentage refers to the number of pretreated patients. ^b^Patients in the OLE received 600 mg OCR every 6M. ^c^The first OCR dose was given as 2 x 1000 mg infusions with a 14-day interval. ^d^At Week 24, OCR 2000 mg was reduced to 1000 mg. ^e^The first OCR dose was given as 2 x 300 mg infusions with a 14-day interval. ^f^At Week 24, patients in the placebo and IFN β-1a groups received OCR 600 mg. ^g^All doses of OCR were given as 2 x 300 mg infusions. ^h^Optional infusion at Week 96 (dose 5; not included in safety analysis); eligible patients could participate in the CHORDS extension substudy, where they received a 600 mg infusion of OCR over a reduced infusion time of approximately 2 hours. ^i^Estimated enrollment. ^j^An initial dose of 2 x 300 mg infusions separated by 14 days (on Days 1 and 15), and then 600 mg every 24 weeks for the remainder of the study treatment period (approximately 192 weeks).

6M, 6 months; ARR, annualized relapse rate; CDP, confirmed disability progression; CSF, cerebrospinal fluid; CTCAE, Common Terminology Criteria for Adverse Events; DMT, disease-modifying therapy; Gd, gadolinium; IFN, interferon; MS, multiple sclerosis; NCI, National Cancer Institute; NEDA, no evidence of disease activity; NEP, no evidence of progression; NEPAD, no evidence of progression and no active disease; NfL, neurofilament light; OCR, ocrelizumab; OLE, open-label extension; PMS, progressive multiple sclerosis; PPMS, primary progressive multiple sclerosis; PY, patient years; RMS, relapsing multiple sclerosis; RRMS, relapsing-remitting multiple sclerosis; SPMS, secondary progressive multiple sclerosis.

# Table S2. Standard criteria for determining seriousness and severity of AEs.

# Criteria for seriousness

| **Criteria^a^** | **Definition** |
| --- | --- |
| **1** | Requires or prolongs hospitalization |
| **2** | Other medically important serious event |
| **3** | Persistent or significant disability/incapacity |
| **4** | Congenital anomaly or birth defect |
| **5** | Is life-threatening |
| **6** | Results in death |

# Severity grades

| **Grade** | **Definition** |
| --- | --- |
| **Grade I** | **Mild**: Asymptomatic or mild symptoms; clinical or diagnostic observations only; intervention not indicated |
| **Grade II** | **Moderate**: Minimal, local, or non-invasive intervention indicated; limiting age-appropriate instrumental activities of daily living (preparing meals, shopping for groceries or clothes, using the telephone, managing money, etc.) |
| **Grade III** | **Severe or medically significant but not immediately life-threatening:** Hospitalization or prolongation of hospitalization indicated; disabling; limiting self-care activities of daily living (bathing, dressing and undressing, feeding self, using the toilet, taking medications, and not bedridden) |
| **Grade IV** | **Life-threatening consequences:** Urgent intervention indicated |
| **Grade V** | **Death** related to AE (not applicable for all AEs) |

(A) Definitions for classification of AEs as serious.^1^ (B) Standard criteria for determining severity^2^ of AEs (CTCAE v5.0, 2017).

^a^AEs were defined as serious if they met one or more of the listed criteria.

AE, adverse event; CTCAE, Common Terminology Criteria for Adverse Events.

# Table S3. Comorbid conditions included in Charlson–Deyo and the Elixhauser comorbidity indices, with corresponding MedDRA classes.^3,4^

| **Charlson–Deyo and Elixhauser list of comorbidities** | **MedDRA HLGT or HLT or SMQ or SOC** | **Groups** |
| --- | --- | --- |
| Myocardial infarction | **HLGT**: Coronary artery disorders  **HLGT**: Cardiac valve disorders | Cardiovascular disorders |
| Congestive heart failure | **HLGT**: Heart failures |  |
| Peripheral vascular disease | **HLT**: Peripheral vasoconstriction, necrosis, and vascular insufficiency |  |
| Hypertension | **HLT**: Vascular hypertensive disorders NEC |  |
| Cerebrovascular disease | **HLT**: Central nervous system vascular disorders NEC | Cerebrovascular disorders |
| Dementia | **HLGT**: Dementia and amnestic conditions | Dementia |
| Chronic pulmonary disease | **HLGT**: Bronchial disorders NEC (excluding neoplasms) | Chronic pulmonary disease |
| Rheumatologic disease | **HLGT**: Connective tissue disorders (excluding congenital)  **HLT**: Immune and associated conditions NEC  **HLT**: Psoriatic conditions | Rheumatologic and systemic autoimmune disorders |
| Peptic ulcer disease | **HLGT**: Gastrointestinal ulceration and perforation  **HLT**: Malabsorption syndromes (except tropical sprue and Whipple’s disease) | Gastrointestinal and liver disorders |
| Mild liver disease | **HLT**: Hepatocellular damage and hepatitis NEC  **HLT**: Hepatic fibrosis and cirrhosis |  |
| Moderate or severe liver disease |  |  |
| Diabetes without chronic complication | **HLT**: Diabetes mellitus (including subtypes)  **HLGT**: Diabetic complications | Diabetes |
| Diabetes with chronic complication |  |  |
| Hemiplegia or paraplegia | Not included as this is accounted for by EDSS in the model | N/A |
| Renal disease | **HLT**: Renal failure and impairment  **HLT**: Renal failure complications | Renal disorders |
| Acquired immune deficiency syndrome | Not included as HIV infection was an exclusion criterion | N/A |
| Any malignancy without metastasis | **SMQ**: Malignancy-related conditions | Malignancies |
| Leukemia |  |  |
| Lymphoma |  |  |
| Metastatic solid tumor |  |  |
| N/A | **HLT**: Bladder reflux conditions  **HLT:** Myoneurogenic bladder disorders  **HLGT**: Ureteric disorders  **HLGT**: Urethral disorders (except urethral infections and inflammations and urethral neoplasms)  **HLGT**: Urolithiases  **HLT**: Bladder and urethral symptoms  **HLT**: Therapeutic bladder catheterization | Urinary tract disorders |
| N/A | **HLGT**: Depressed mood disorders and disturbances | Depression^a^ |
| N/A | **SOC**: Infections and infestations | Previous infections^b^ |

A modified version of the Charlson–Deyo and Elixhauser comorbidity indices is presented here. Urinary tract disorders, depression, and previous infections (except for HIV) are not part of these indices, but these are of high relevance in MS.
^a^Depression has been shown to confer an increased risk of infections.^5^
^b^Only events before initiation of ocrelizumab therapy were considered.

EDSS, Expanded Disability Status Scale; HIV, human immunodeficiency virus; HLGT, High Level Group Term; HLT, High Level Term;
MedDRA, Medical Dictionary for Regulatory Activities; MS, multiple sclerosis; N/A, not applicable; NEC, Not Elsewhere Classified;
SMQ, Standardised MedDRA Queries; SOC, System Organ Class.

# Table S4. Rates of infections and SIs according to the presence of comorbidities at the last known follow-up.^a^

# RMS (OPERA)

|  | **All infections** | | | **SIs** | | |
| --- | --- | --- | --- | --- | --- | --- |
| **Characteristic** | **Infections, *n*** | **Patients,**  ***n*** | **Rates per 100 PY (95% CI)** | **SIs, *n*** | **Patients,  *n*** | **Rates per 100 PY**  **(95% CI)** |
| **Overall** | 7141 | 1141 | 66.07 (64.54–67.62) | 190 | 144 | 1.76 (1.52–2.03) |
| **Number of comorbidities** |  |  |  |  |  |  |
| **0** | 1478 | 325 | 40.60 (38.55–42.72) | 36 | 30 | 0.99 (0.69–1.37) |
| **1** | 2111 | 364 | 63.59 (60.91–66.36) | 49 | 43 | 1.48 (1.09–1.95) |
| **≥2** | 3552 | 452 | 92.30 (89.29–95.39) | 105 | 71 | 2.73 (2.23–3.30) |
| **Cardiovascular disorders** |  |  |  |  |  |  |
| Yes | 1552 | 227 | 73.50 (69.88–77.25) | 52 | 40 | 2.46 (1.84–3.23) |
| No | 5589 | 914 | 64.26 (62.59–65.97) | 138 | 104 | 1.59 (1.33–1.87) |
| **Cerebrovascular disorders** |  |  |  |  |  |  |
| Yes | 21 | 3 | 68.09 (42.15–104.08) | 2 | 2 | 6.48 (0.79–23.42) |
| No | 7120 | 1138 | 66.06 (64.54–67.61) | 188 | 142 | 1.74 (1.50–2.01) |
| **Chronic pulmonary disease** |  |  |  |  |  |  |
| Yes | 1005 | 115 | 99.54 (93.48–105.89) | 23 | 20 | 2.28 (1.44–3.42) |
| No | 6136 | 1026 | 62.62 (61.06–64.21) | 167 | 124 | 1.70 (1.46–1.98) |
| **Rheumatologic/autoimmune disorders** |  |  |  |  |  |  |
| Yes | 509 | 48 | 131.35 (120.19–143.27) | 9 | 8 | 2.32 (1.06–4.41) |
| No | 6632 | 1093 | 63.64 (62.12–65.19) | 181 | 136 | 1.74 (1.49–2.01) |
| **Gastrointestinal/liver disorders** |  |  |  |  |  |  |
| Yes | 378 | 39 | 92.05 (83.00–101.81) | 8 | 5 | 1.95 (0.84–3.84) |
| No | 6763 | 1102 | 65.04 (63.50–66.61) | 182 | 139 | 1.75 (1.51–2.02) |
| **Diabetes** |  |  |  |  |  |  |
| Yes | 344 | 40 | 89.61 (80.39–99.60) | 21 | 13 | 5.47 (3.39–8.36) |
| No | 6797 | 1101 | 65.20 (63.66–66.77) | 169 | 131 | 1.62 (1.39–1.88) |
| **Renal disorders** |  |  |  |  |  |  |
| Yes | 43 | 4 | 144.05 (104.25–194.03) | 4 | 3 | 13.40 (3.65–34.31) |
| No | 7098 | 1137 | 65.85 (64.33–67.40) | 186 | 141 | 1.73 (1.49–1.99) |
| **Malignancies** |  |  |  |  |  |  |
| Yes | 13 | 4 | 40.52 (21.57–69.29) | 0 | 0 | 0.00 (NE–11.50) |
| No | 7128 | 1137 | 66.14 (64.62–67.70) | 190 | 144 | 1.76 (1.52–2.03) |
| **Urinary tract disorders** |  |  |  |  |  |  |
| Yes | 2093 | 236 | 101.65 (97.34–106.10) | 63 | 40 | 3.06 (2.35–3.91) |
| No | 5048 | 905 | 57.69 (56.11–59.31) | 127 | 104 | 1.45 (1.21–1.73) |
| **Depression** |  |  |  |  |  |  |
| Yes | 2749 | 389 | 84.95 (81.80–88.18) | 68 | 51 | 2.10 (1.63–2.66) |
| No | 4392 | 752 | 58.00 (56.30–59.74) | 122 | 93 | 1.61 (1.34–1.92) |
| **Previous infections, OCR start** |  |  |  |  |  |  |
| Yes | 2878 | 419 | 84.35 (81.30–87.49) | 80 | 55 | 2.34 (1.86–2.92) |
| No | 4263 | 722 | 57.63 (55.92–59.39) | 110 | 89 | 1.49 (1.22–1.79) |

# PPMS (ORATORIO)

|  | **All infections** | | | **SIs** | | |
| --- | --- | --- | --- | --- | --- | --- |
| **Characteristic** | **Infections, *n*** | **Patients,**  ***n*** | **Rates per 100 PY (95% CI)** | **SIs, *n*** | **Patients,  *n*** | **Rates per 100 PY**  **(95% CI)** |
| **Overall** | 3288 | 531 | 69.76 (67.40–72.19) | 205 | 121 | 4.35 (3.77–4.99) |
| **Number of comorbidities** |  |  |  |  |  |  |
| **0** | 615 | 134 | 45.10 (41.61–48.81) | 36 | 19 | 2.64 (1.85–3.65) |
| **1** | 736 | 125 | 64.95 (60.34–69.81) | 39 | 28 | 3.44 (2.45–4.70) |
| **≥2** | 1937 | 272 | 87.40 (83.55–91.38) | 130 | 74 | 5.87 (4.90–6.97) |
| **Cardiovascular disorders** |  |  |  |  |  |  |
| Yes | 1133 | 154 | 84.76 (79.89–89.84) | 79 | 42 | 5.91 (4.68–7.37) |
| No | 2155 | 377 | 63.83 (61.16–66.58) | 126 | 79 | 3.73 (3.11–4.44) |
| **Cerebrovascular disorders** |  |  |  |  |  |  |
| Yes | 28 | 4 | 112.25 (74.59–162.23) | 2 | 2 | 8.02 (0.97–28.96) |
| No | 3260 | 527 | 69.54 (67.17–71.97) | 203 | 119 | 4.33 (3.75–4.97) |
| **Chronic pulmonary disease** |  |  |  |  |  |  |
| Yes | 363 | 43 | 98.45 (88.58–109.12) | 16 | 12 | 4.34 (2.48–7.05) |
| No | 2925 | 488 | 67.33 (64.91–69.81) | 189 | 109 | 4.35 (3.75–5.02) |
| **Rheumatologic/autoimmune disorders** |  |  |  |  |  |  |
| Yes | 245 | 29 | 92.06 (80.90–104.34) | 13 | 5 | 4.89 (2.60–8.35) |
| No | 3043 | 502 | 68.43 (66.02–70.90) | 192 | 116 | 4.32 (3.73–4.97) |
| **Gastrointestinal/liver disorders** |  |  |  |  |  |  |
| Yes | 140 | 24 | 58.33 (49.06–68.83) | 4 | 4 | 1.67 (0.45–4.27) |
| No | 3148 | 507 | 70.38 (67.94–72.88) | 201 | 117 | 4.49 (3.89–5.16) |
| **Diabetes** |  |  |  |  |  |  |
| Yes | 286 | 38 | 90.60 (80.40–101.73) | 19 | 14 | 6.02 (3.62–9.40) |
| No | 3002 | 493 | 68.27 (65.85–70.75) | 186 | 107 | 4.23 (3.64–4.88) |
| **Renal disorders** |  |  |  |  |  |  |
| Yes | 82 | 8 | 180.51 (143.57–224.06) | 5 | 4 | 11.01 (3.57–25.69) |
| No | 3206 | 523 | 68.69 (66.33–71.11) | 200 | 117 | 4.28 (3.71–4.92) |
| **Malignancies** |  |  |  |  |  |  |
| Yes | 17 | 2 | 78.29 (45.61–125.35) | 1 | 1 | 4.61 (0.12–25.66) |
| No | 3271 | 529 | 69.72 (67.35–72.16) | 204 | 120 | 4.35 (3.77–4.99) |
| **Urinary tract disorders** |  |  |  |  |  |  |
| Yes | 1198 | 150 | 94.81 (89.51–100.33) | 96 | 48 | 7.60 (6.15–9.28) |
| No | 2090 | 381 | 60.59 (58.02–63.24) | 109 | 73 | 3.16 (2.59–3.81) |
| **Depression** |  |  |  |  |  |  |
| Yes | 1187 | 173 | 88.33 (83.38–93.50) | 81 | 47 | 6.03 (4.79–7.49) |
| No | 2101 | 358 | 62.36 (59.72–65.08) | 124 | 74 | 3.68 (3.06–4.39) |
| **Previous infections, OCR start** |  |  |  |  |  |  |
| Yes | 1230 | 198 | 83.31 (78.71–88.09) | 83 | 51 | 5.62 (4.48–6.97) |
| No | 2058 | 333 | 63.59 (60.87–66.39) | 122 | 70 | 3.77 (3.13–4.50) |

Rates of infections and SIs according to the presence of comorbidities at the last known follow-up in patients from the pivotal trials and respective open-label extensions: OPERA (RMS) and ORATORIO (PPMS). The table suggests that comorbidities are best included in the multivariate model as categorical variables (0, 1, and ≥2 comorbidities). The risk associated with each comorbidity category was analyzed in univariate analyses (Supplementary Figures 6 and 7). Investigator text for AEs encoded using MedDRA version 22.1. Multiple occurrences of the same AE in one patient were counted multiple times. 95% CIs were calculated using an exact method based on the Poisson distribution.
^a^Last known follow-up was at time of study discontinuation, December 31, 2022.

AE, adverse event; CI, confidence interval; MedDRA, Medical Dictionary for Regulatory Activities; NE, non-estimable; OCR, ocrelizumab; PPMS, primary progressive multiple sclerosis; PY, patient years;

RMS, relapsing multiple sclerosis; SI, serious infection.

# Table S5. Rates of all infections and SIs distributed according to baseline characteristics.

# A. RMS (OPERA)

|  | **All infections** | | | **SIs** | | |
| --- | --- | --- | --- | --- | --- | --- |
| **Baseline characteristics** | **Infections, *n*** | **Patients with event, *n*** | **Rate per 100 PY (95% CI)** | **SIs, *n*** | **Patients with event, *n*** | **Rate per 100 PY**  **(95% CI)** |
| **Overall (*N* = 1448 patients)** | 7141 | 1141 | 66.07 (64.54–67.62) | 190 | 144 | 1.76 (1.52–2.03) |
| **Age, years** |  |  |  |  |  |  |
| <40 | 4002 | 638 | 67.08 (65.01–69.19) | 94 | 77 | 1.58 (1.27–1.93) |
| 40–59 | 3139 | 503 | 64.82 (62.58–67.13) | 96 | 67 | 1.98 (1.61–2.42) |
| **Sex** |  |  |  |  |  |  |
| Female | 5256 | 776 | 75.70 (73.67–77.78) | 112 | 84 | 1.61 (1.33–1.94) |
| Male | 1885 | 365 | 48.76 (46.59–51.01) | 78 | 60 | 2.02 (1.59–2.52) |
| **BMI, kg/m^2^** |  |  |  |  |  |  |
| Underweight <18.5 | 305 | 44 | 70.51 (62.82–78.88) | 17 | 7 | 3.93 (2.29–6.29) |
| Normal weight 18.5 to <25.0 | 2960 | 519 | 59.06 (56.95–61.22) | 81 | 65 | 1.62 (1.28–2.01) |
| Overweight 25.0 to <30.0 | 2052 | 303 | 70.51 (67.49–73.62) | 47 | 40 | 1.61 (1.19–2.15) |
| Obese ≥30.0 | 1713 | 263 | 73.25 (69.82–76.80) | 42 | 30 | 1.80 (1.29–2.43) |
| Missing | 111 | 12 | 96.67 (79.53–116.42) | 3 | 2 | 2.61 (0.54–7.64) |
| **Region** |  |  |  |  |  |  |
| USA | 2020 | 306 | 79.08 (75.67–82.61) | 49 | 37 | 1.92 (1.42–2.54) |
| ROW | 5121 | 835 | 62.04 (60.35–63.76) | 141 | 107 | 1.71 (1.44–2.01) |
| **Race** |  |  |  |  |  |  |
| White | 6549 | 1032 | 66.56 (64.96–68.20) | 175 | 133 | 1.78 (1.52–2.06) |
| Black/African American | 188 | 47 | 48.44 (41.76–55.88) | 3 | 2 | 0.77 (0.16–2.26) |
| Asian | 22 | 3 | 63.07 (39.53–95.49) | 0 | 0 | 0.00 (NE–10.58) |
| Other | 382 | 59 | 69.83 (63.00–77.20) | 12 | 9 | 2.19 (1.13–3.83) |
| **Comorbidities** |  |  |  |  |  |  |
| 0 | 2724 | 488 | 52.42 (50.47–54.42) | 75 | 63 | 1.44 (1.14–1.81) |
| 1 | 2492 | 390 | 70.85 (68.10–73.69) | 61 | 43 | 1.73 (1.33–2.23) |
| ≥2 | 1925 | 263 | 91.90 (87.84–96.10) | 54 | 38 | 2.58 (1.94–3.36) |
| **EDSS** |  |  |  |  |  |  |
| <3.0 | 4338 | 661 | 70.18 (68.11–72.30) | 92 | 77 | 1.49 (1.20–1.83) |
| 3.0–6.0 | 2750 | 473 | 60.43 (58.19–62.73) | 97 | 66 | 2.13 (1.73–2.60) |
| >6.0 | 53 | 7 | 69.21 (51.84–90.53) | 1 | 1 | 1.31 (0.03–7.28) |
| **Disease duration, years** |  |  |  |  |  |  |
| <5 | 3500 | 534 | 69.55 (67.27–71.90) | 81 | 64 | 1.61 (1.28–2.00) |
| 5–10 | 1749 | 316 | 58.54 (55.83–61.35) | 40 | 34 | 1.34 (0.96–1.82) |
| >10 | 1892 | 291 | 67.84 (64.82–70.97) | 69 | 46 | 2.47 (1.92–3.13) |
| **Relapse at study start** |  |  |  |  |  |  |
| None | 234 | 36 | 65.35 (57.25–74.28) | 6 | 4 | 1.68 (0.61–3.65) |
| ≥1 | 6907 | 1105 | 66.09 (64.54–67.67) | 184 | 140 | 1.76 (1.52–2.03) |
| **Relapses in year before OCR start** |  |  |  |  |  |  |
| Yes | 5069 | 732 | 71.71 (69.75–73.71) | 133 | 105 | 1.88 (1.58–2.23) |
| No | 2072 | 409 | 55.40 (53.04–57.84) | 57 | 39 | 1.52 (1.15–1.97) |
| **Previous treatment with DMT**  **(prior to OCR start)^a^** |  |  |  |  |  |  |
| Yes | 3987 | 679 | 64.82 (62.83–66.87) | 126 | 90 | 2.05 (1.71–2.44) |
| No | 3154 | 462 | 67.71 (65.37–70.11) | 64 | 54 | 1.37 (1.06–1.75) |
| **Previous treatment with DMT**  **(study start)** |  |  |  |  |  |  |
| Yes | 2171 | 314 | 76.23 (73.06–79.51) | 67 | 44 | 2.35 (1.82–2.99) |
| No | 4970 | 827 | 62.43 (60.71–64.19) | 123 | 100 | 1.55 (1.28–1.84) |
| **IgG, g/L (LLN = 5.65 g/L)** |  |  |  |  |  |  |
| <5.65 | 31 | 6 | 55.38 (37.63–78.61) | 2 | 1 | 3.57 (0.43–12.91) |
| ≥5.65 | 7107 | 1133 | 66.22 (64.69–67.77) | 187 | 142 | 1.74 (1.50–2.01) |
| Q1 (≤9.16) | 2044 | 289 | 77.12 (73.81–80.54) | 51 | 38 | 1.92 (1.43–2.53) |
| Q2 (9.16–10.70) | 1836 | 298 | 65.35 (62.39–68.41) | 59 | 44 | 2.10 (1.60–2.71) |
| Q3 (10.70–12.50) | 1650 | 282 | 60.48 (57.60–63.47) | 41 | 33 | 1.50 (1.08–2.04) |
| Q4 (>12.50) | 1608 | 270 | 61.82 (58.84–64.92) | 38 | 28 | 1.46 (1.03–2.01) |
| **IgM, g/L (LLN = 0.4 g/L)** |  |  |  |  |  |  |
| <0.4 | 25 | 7 | 44.40 (28.73–65.54) | 1 | 1 | 1.78 (0.04–9.89) |
| ≥0.4 | 7113 | 1132 | 66.27 (64.74–67.83) | 188 | 142 | 1.75 (1.51–2.02) |
| Q1 (≤0.86) | 1742 | 288 | 64.75 (61.74–67.86) | 51 | 35 | 1.90 (1.41–2.49) |
| Q2 (0.86–1.21) | 2008 | 290 | 73.21 (70.04–76.48) | 56 | 42 | 2.04 (1.54–2.65) |
| Q3 (1.21–1.64) | 1767 | 289 | 64.95 (61.95–68.05) | 35 | 31 | 1.29 (0.90–1.79) |
| Q4 (>1.64) | 1621 | 272 | 61.52 (58.56–64.59) | 47 | 35 | 1.78 (1.31–2.37) |
| **Lymphocytes, cells x 10^9^/L (LLN = 0.91x10^9^/L)** |  |  |  |  |  |  |
| <0.91 | 357 | 65 | 60.16 (54.08–66.73) | 15 | 9 | 2.53 (1.41–4.17) |
| ≥0.91 | 6781 | 1073 | 66.70 (65.12–68.31) | 175 | 135 | 1.72 (1.48–2.00) |
| Q1 (≤1.30) | 1770 | 289 | 65.73 (62.71–68.87) | 53 | 39 | 1.97 (1.47–2.57) |
| Q2 (1.30–1.66) | 1669 | 277 | 62.55 (59.58–65.62) | 29 | 25 | 1.09 (0.73–1.56) |
| Q3 (1.66–2.05) | 1826 | 284 | 66.86 (63.83–70.00) | 64 | 45 | 2.34 (1.80–2.99) |
| Q4 (>2.05) | 1873 | 288 | 70.22 (67.07–73.47) | 44 | 35 | 1.65 (1.20–2.21) |
| **Neutrophils, cells x 10^9^/L (LLN = 1.96x10^9^/L)** |  |  |  |  |  |  |
| <1.96 | 530 | 89 | 62.34 (57.14–67.88) | 22 | 12 | 2.59 (1.62–3.92) |
| ≥1.96 | 6608 | 1049 | 66.68 (65.09–68.31) | 168 | 132 | 1.70 (1.45–1.97) |
| Q1 (≤2.70) | 1630 | 281 | 61.71 (58.75–64.78) | 48 | 34 | 1.82 (1.34–2.41) |
| Q2 (2.70–3.59) | 1794 | 288 | 64.93 (61.96–68.00) | 32 | 24 | 1.16 (0.79–1.63) |
| Q3 (3.59–4.61) | 1944 | 290 | 71.31 (68.17–74.55) | 62 | 50 | 2.27 (1.74–2.92) |
| Q4 (>4.61) | 1770 | 279 | 67.32 (64.22–70.53) | 48 | 36 | 1.83 (1.35–2.42) |
| **B cells, cells/µL (LLN = 80 cells/µL)** |  |  |  |  |  |  |
| <80 | 223 | 34 | 67.32 (58.78–76.76) | 6 | 5 | 1.81 (0.66–3.94) |
| ≥80 | 6604 | 1056 | 66.41 (64.82–68.03) | 176 | 132 | 1.77 (1.52–2.05) |

# B. PPMS (ORATORIO)

|  | **All infections** | | | **SIs** | | |
| --- | --- | --- | --- | --- | --- | --- |
| **Baseline characteristics** | **Infections, *n*** | **Patients with event, *n*** | **Rate per 100 PY**  **(95% CI)** | **SIs, *n*** | **Patients with event, *n*** | **Rate per 100 PY**  **(95% CI)** |
| **Overall (*N* = 644 patients)** | 3288 | 531 | 69.76 (67.40–72.19) | 205 | 121 | 4.35 (3.77–4.99) |
| **Age, years** |  |  |  |  |  |  |
| <40 | 634 | 111 | 60.05 (55.47–64.91) | 43 | 25 | 4.07 (2.95–5.49) |
| 40–59 | 2654 | 420 | 72.57 (69.83–75.38) | 162 | 96 | 4.43 (3.77–5.17) |
| **Sex** |  |  |  |  |  |  |
| Female | 1906 | 276 | 82.61 (78.94–86.40) | 87 | 56 | 3.77 (3.02–4.65) |
| Male | 1382 | 255 | 57.45 (54.46–60.56) | 118 | 65 | 4.90 (4.06–5.87) |
| **BMI, kg/m^2^** |  |  |  |  |  |  |
| Underweight <18.5 | 175 | 22 | 102.37 (87.76–118.71) | 8 | 2 | 4.68 (2.02–9.22) |
| Normal weight 18.5 to <25.0 | 1596 | 279 | 63.34 (60.27–66.53) | 75 | 57 | 2.98 (2.34–3.73) |
| Overweight 25.0 to <30.0 | 955 | 146 | 74.25 (69.61–79.11) | 81 | 37 | 6.30 (5.00–7.83) |
| Obese ≥30.0 | 557 | 83 | 76.75 (70.51–83.40) | 41 | 25 | 5.65 (4.05–7.66) |
| Missing | 5 | 1 | 47.25 (15.34–110.27) | 0 | 0 | 0.00 (NE–34.86) |
| **Region** |  |  |  |  |  |  |
| USA | 563 | 76 | 97.51 (89.62–105.91) | 48 | 24 | 8.31 (6.13–11.02) |
| ROW | 2725 | 455 | 65.89 (63.44–68.41) | 157 | 97 | 3.80 (3.23–4.44) |
| **Race** |  |  |  |  |  |  |
| White | 3099 | 500 | 69.77 (67.34–72.27) | 193 | 115 | 4.35 (3.75–5.00) |
| Black/African American | 45 | 8 | 69.35 (50.58–92.79) | 3 | 2 | 4.62 (0.95–13.51) |
| Other | 142 | 22 | 70.79 (59.63–83.44) | 8 | 3 | 3.99 (1.72–7.86) |
| Unknown | 2 | 1 | 33.20 (4.02–119.95) | 1 | 1 | 16.60 (0.42–92.50) |
| **Comorbidities** |  |  |  |  |  |  |
| 0 | 1137 | 197 | 56.49 (53.26–59.87) | 63 | 36 | 3.13 (2.41–4.00) |
| 1 | 1054 | 168 | 72.98 (68.64–77.52) | 60 | 39 | 4.15 (3.17–5.35) |
| ≥2 | 1097 | 166 | 87.33 (82.24–92.65) | 82 | 46 | 6.53 (5.19–8.10) |
| **EDSS** |  |  |  |  |  |  |
| <3.0 | 83 | 12 | 91.85 (73.16–113.86) | 3 | 3 | 3.32 (0.68–9.70) |
| 3.0–6.0 | 2685 | 442 | 67.46 (64.93–70.06) | 132 | 86 | 3.32 (2.77–3.93) |
| >6.0 | 495 | 76 | 78.13 (71.39–85.32) | 69 | 31 | 10.89 (8.47–13.78) |
| Missing | 25 | 1 | 285.80 (184.95–421.89) | 1 | 1 | 11.43 (0.29–63.69) |
| **Disease duration** |  |  |  |  |  |  |
| <5 | 1078 | 165 | 70.27 (66.14–74.60) | 52 | 40 | 3.39 (2.53–4.45) |
| 5–10 | 1494 | 240 | 72.72 (69.08–76.51) | 109 | 51 | 5.31 (4.36–6.40) |
| >10 | 668 | 115 | 68.59 (63.49–74.00) | 41 | 27 | 4.21 (3.02–5.71) |
| Missing | 48 | 11 | 31.81 (23.45–42.17) | 3 | 3 | 1.99 (0.41–5.81) |
| **Relapses before OCR start** |  |  |  |  |  |  |
| Yes | 81 | 14 | 78.63 (62.45–97.73) | 4 | 2 | 3.88 (1.06–9.94) |
| No | 3207 | 517 | 69.57 (67.18–72.02) | 201 | 119 | 4.36 (3.78–5.01) |
| **Relapses in year before OCR start** |  |  |  |  |  |  |
| Yes | 39 | 5 | 128.89 (91.65–176.19) | 3 | 1 | 9.91 (2.04–28.97) |
| No | 3249 | 526 | 69.38 (67.02–71.81) | 202 | 120 | 4.31 (3.74–4.95) |
| **Previous treatment with DMT**  **(prior – OCR start)** |  |  |  |  |  |  |
| Yes | 324 | 59 | 63.49 (56.76–70.79) | 27 | 19 | 5.29 (3.49–7.70) |
| No | 2964 | 472 | 70.53 (68.01–73.11) | 178 | 102 | 4.24 (3.64–4.91) |
| **Previous treatment with DMT**  **(study start)** |  |  |  |  |  |  |
| Yes | 320 | 58 | 64.02 (57.19–71.43) | 27 | 19 | 5.40 (3.56–7.86) |
| No | 2968 | 473 | 70.45 (67.93–73.03) | 178 | 102 | 4.22 (3.63–4.89) |
| **IgG, g/L (LLN = 5.65 g/L)** |  |  |  |  |  |  |
| <5.65 | 10 | 2 | 67.34 (32.29–123.84) | 0 | 0 | 0.00 (NE–24.84) |
| ≥5.65 | 3276 | 528 | 69.94 (67.56–72.37) | 205 | 121 | 4.38 (3.80–5.02) |
| Q1 (≤8.75) | 907 | 130 | 77.46 (72.50–82.67) | 52 | 34 | 4.44 (3.32–5.82) |
| Q2 (8.75–10.30) | 951 | 147 | 80.21 (75.19–85.48) | 69 | 35 | 5.82 (4.53–7.37) |
| Q3 (10.30–11.90) | 712 | 132 | 63.57 (58.99–68.42) | 51 | 31 | 4.55 (3.39–5.99) |
| Q4 (>11.90) | 716 | 121 | 58.56 (54.35–63.01) | 33 | 21 | 2.70 (1.86–3.79) |
| **IgM, g/L (LLN = 0.4 g/L)** |  |  |  |  |  |  |
| <0.4 | 10 | 3 | 42.62 (20.44–78.38) | 0 | 0 | 0.00 (NE–15.72) |
| ≥0.4 | 3276 | 527 | 70.06 (67.69–72.51) | 205 | 121 | 4.38 (3.80–5.03) |
| Q1 (≤0.83) | 901 | 132 | 77.14 (72.18–82.34) | 88 | 37 | 7.53 (6.04–9.28) |
| Q2 (0.83–1.18) | 808 | 133 | 70.28 (65.52–75.30) | 32 | 26 | 2.78 (1.90–3.93) |
| Q3 (1.18–1.62) | 881 | 141 | 73.42 (68.66–78.44) | 46 | 32 | 3.83 (2.81–5.11) |
| Q4 (>1.62) | 696 | 124 | 58.90 (54.61–63.45) | 39 | 26 | 3.30 (2.35–4.51) |
| **Lymphocytes, cells x10^9^/L (LLN = 0.91x10^9^/L)** |  |  |  |  |  |  |
| <0.91 | 68 | 8 | 97.21 (75.48–123.23) | 1 | 1 | 1.43 (0.04–7.96) |
| ≥0.91 | 3220 | 523 | 69.35 (66.97–71.79) | 204 | 120 | 4.39 (3.81–5.04) |
| Q1 (≤1.42) | 961 | 132 | 80.78 (75.76–86.06) | 67 | 33 | 5.63 (4.36–7.15) |
| Q2 (1.42–1.81) | 759 | 127 | 62.57 (58.20–67.19) | 46 | 26 | 3.79 (2.78–5.06) |
| Q3 (1.81–2.21) | 829 | 139 | 69.18 (64.55–74.05) | 47 | 27 | 3.92 (2.88–5.22) |
| Q4 (>2.21) | 739 | 133 | 66.45 (61.74–71.42) | 45 | 35 | 4.05 (2.95–5.41) |
| **Neutrophils, cells x10^9^/L (LLN = 1.96x10^9^/L)** |  |  |  |  |  |  |
| <1.96 | 52 | 7 | 76.51 (57.14–100.33) | 2 | 2 | 2.94 (0.36–10.63) |
| ≥1.96 | 3236 | 524 | 69.66 (67.28–72.11) | 203 | 119 | 4.37 (3.79–5.01) |
| Q1 (≤3.34) | 941 | 134 | 75.52 (70.77–80.50) | 55 | 31 | 4.41 (3.33–5.75) |
| Q2 (3.34–4.23) | 830 | 139 | 66.92 (62.45–71.63) | 47 | 28 | 3.79 (2.78–5.04) |
| Q3 (4.23–5.43) | 722 | 125 | 67.50 (62.67–72.61) | 55 | 30 | 5.14 (3.87–6.69) |
| Q4 (>5.43) | 795 | 133 | 68.71 (64.01–73.65) | 48 | 32 | 4.15 (3.06–5.50) |
| **B cells, cells/µL (LLN = 80 cells/µL)** |  |  |  |  |  |  |
| <80 | 155 | 23 | 60.90 (51.69–71.28) | 8 | 5 | 3.14 (1.36–6.19) |
| ≥80 | 2991 | 484 | 70.50 (68.00–73.07) | 193 | 112 | 4.55 (3.93–5.24) |

^a^Including patients in OPERA who started in the IFN arm of the study. Rates of infections and SIs stratified by demographics, disease characteristics, and immunologic status at baseline in patients from the pivotal trials and respective open-label extensions: OPERA (RMS) and ORATORIO (PPMS). For more details on how the results below were used to inform the univariate and multivariate models, please see Methods. Multiple occurrences of the same adverse event in one patient were counted multiple times. For patients who switched from IFN or placebo to OCR at the end of the double-blind period, age, EDSS, comorbidity, disease duration, and all laboratory data were re-derived at the beginning of the open-label extension. LLNs defined according to the reference values of the central laboratory.

BMI, body mass index; CI, confidence interval; DMT, disease-modifying therapy; EDSS, Expanded Disability Status Scale; IFN, interferon; Ig, immunoglobulin; LLN, lower limit of normal; NE, non-existing;

OCR, ocrelizumab; PPMS, primary progressive multiple sclerosis; PY, patient years; Q, quartile; RMS, relapsing multiple sclerosis; ROW, rest of the world; SI, serious infection.

# Table S6. Yearly rates of infections, excluding COVID-19.

# RMS (*n* = 4558)

|  | **Treatment duration** | | | | | | | | | | | |
| --- | --- | --- | --- | --- | --- | --- | --- | --- | --- | --- | --- | --- |
| **Characteristics** | **Year 1** | **Year 2** | **Year 3** | **Year 4** | **Year 5** | **Year 6** | **Year 7** | **Year 8** | **Year 9** | **Year 10** | **Year 11** | **Year 12** |
| **Patients, *n*** | 4558 | 4360 | 3435 | 2979 | 1881 | 1351 | 1198 | 1131 | 910 | 611 | 332 | 79 |
| **PY** | 4495.9 | 4132.3 | 3168.5 | 2590.1 | 1648.4 | 1238.6 | 1160.5 | 1080.6 | 738.3 | 529.1 | 177.0 | 70.9 |
| **Rates per 100 PY (95% CIs)** | 92.28  (89.50–95.14) | 67.28  (64.80–69.82) | 58.26  (55.63–60.98) | 54.55  (51.75–57.47) | 57.57  (53.97–61.35) | 65.40  (60.97–70.06) | 57.73  (53.44–62.27) | 57.56  (53.12–62.26) | 45.51  (40.77–50.65) | 50.65  (44.77–57.09) | 38.41  (29.83–48.69) | 21.16  (11.84–34.90) |

# PMS (*n* = 1597)

|  | **Treatment duration** | | | | | | | | | | | |
| --- | --- | --- | --- | --- | --- | --- | --- | --- | --- | --- | --- | --- |
| **Characteristics** | **Year 1** | **Year 2** | **Year 3** | **Year 4** | **Year 5** | **Year 6** | **Year 7** | **Year 8** | **Year 9** | **Year 10** | **Year 11** | **Year 12** |
| **Patients, *n*** | 1597 | 1387 | 1199 | 1109 | 583 | 478 | 433 | 322 | 295 | 268 | 200 | 43 |
| **PY** | 1523.9 | 1289.1 | 1157.0 | 886.4 | 518.7 | 456.8 | 405.0 | 301.5 | 282.2 | 250.0 | 111.6 | 7.3 |
| **Rates per 100 PY (95% CIs)** | 67.92  (63.84–72.19) | 50.34  (46.54–54.37) | 51.86  (47.79–56.18) | 53.14  (48.44–58.16) | 70.95  (63.89–78.58) | 65.46  (58.25–73.31) | 84.45  (75.73–93.89) | 79.92  (70.15–90.68) | 60.95  (52.18–70.78) | 65.21  (55.58–76.02) | 70.80  (56.05–88.24) | 82.26  (30.19–179.05) |

CI, confidence interval; COVID-19, coronavirus disease 2019; PMS, progressive multiple sclerosis; PY, patient years; RMS, relapsing multiple sclerosis.

# Table S7. Rates of SIs according to anatomical locations affected, including COVID-19.

| **Types of SIs** | | **Reported terms** | **Events, *n* (%)** | **Rate per 100 PY**  **(95% CI)** | |
| --- | --- | --- | --- | --- | --- |
|  |  |  |  | **RMS** | **PMS** |
| **All SIs (excluding COVID-19)** | |  | **583 (100.0)** | **1.50**  **(1.34–1.68)** | **3.70**  **(3.27–4.17)** |
| **All SIs (including COVID-19)** | |  | **858 (100.0)** | **2.33**  **(2.13–2.55)** | **5.09**  **(4.58–5.64)** |
| **Respiratory tract infections** | **COVID-19^a^** | COVID-19, COVID-19 pneumonia, post-acute COVID-19 syndrome, suspected COVID-19 | 275 (32.1) | 0.83 (0.71–0.96) | 1.39 (1.13–1.69) |
|  | **LRTI** | Pneumonia, bronchitis, pneumonia aspiration, pneumonia bacterial, infective exacerbation of chronic obstructive airways disease, lower respiratory tract infection,  atypical pneumonia, bronchiolitis, pneumocystis jiroveci pneumonia, Escherichia pneumonia, pneumonia hemophilus, pneumonia mycoplasmal, pneumonia viral, pulmonary tuberculosis, tracheobronchitis | 137 (16.0) | 0.40 (0.32–0.50) | **0.72 (0.54–0.95)** |
|  | **URTI** | Chronic sinusitis, sinusitis, acute sinusitis, pharyngitis, upper respiratory tract infection, viral upper respiratory tract infection, cellulitis pharyngeal, fungal pharyngitis, herpes simplex,^b^ peritonsillar abscess | 21 (2.4) | 0.09 (0.05–0.14) | 0.04 (0.01–0.12) |
|  | **Unspecified location** | Influenza, respiratory tract infection, respiratory syncytial virus infection | 9 (1.0) | 0.03  (0.01–0.07) | 0.03 (0.00–0.10) |
| **Urinary tract infections** | | Urinary tract infection, pyelonephritis, pyelonephritis acute, cystitis, bacterial pyelonephritis, Escherichia urinary tract infection, pyelocystitis, pyelonephritis acute, pyuria, renal abscess | 132 (15.4) | 0.24 (0.18–0.32) | **1.13 (0.90–1.40)** |
| **Abdominal and GI infections** | | Appendicitis, gastroenteritis, anal abscess, gastroenteritis viral, diverticulitis, clostridium difficile infection, abdominal abscess, clostridium difficile colitis, large intestine infection, appendicitis perforated, bacterial colitis, complicated appendicitis, enteritis infectious, enterococcal infection, gastroenteritis salmonella, GI infection, gastroenteritis, esophagitis bacterial, oral bacterial infection, parasitic gastroenteritis, perirectal abscess, peritonitis, superinfection fungal | 78 (9.1) | 0.22  (0.16–0.29) | **0.45 (0.30–0.63)** |
| **Skin infections** | | Cellulitis, postoperative wound infection, erysipelas, abscess limb, device-related infection, herpes zoster,^b^ infected dermal cyst, wound infection, acrodermatitis chronica atrophicans, furuncle, herpes zoster disseminated,^b^ impetigo, infected dermal cyst, infective thrombosis, localized infection, periorbital cellulitis, pilonidal disease, pseudomonas infection, skin infection, staphylococcal infection, subcutaneous abscess, varicella,^b^ varicella-zoster virus infection^b^ | 64 (7.5) | 0.15 (0.10–0.21) | **0.45 (0.30–0.63)** |
| **Sepsis** | | Sepsis, urosepsis, neutropenic sepsis, septic shock, candida sepsis, pneumococcal sepsis, post-procedural sepsis, pulmonary sepsis, systemic candida | 54 (6.3) | 0.09 (0.05–0.14) | **0.50 (0.35–0.69)** |
| **Other infections, unspecified location** | | Lyme disease, dengue fever, Borrelia infection, enterovirus infection, Pasteurella infection, typhoid fever, Zika virus, other non-specified infections | 22 (2.6) | 0.07 (0.04–0.12) | 0.10 (0.04–0.20) |
| **Reproductive tract infections** | | Orchitis, pelvic inflammatory disease, salpingitis, epididymitis, fallopian tube abscess, gangrene, genital herpes,^b^ genital herpes simplex,^b^ penile abscess, salpingo-oophoritis, tubo-ovarian abscess, vaginal infection | 22 (2.6) | 0.08 (0.05–0.13) | 0.07  (0.02–0.16) |
| **Central nervous system infections** | | Encephalitis, meningitis (bacterial and viral), herpes simplex meningitis,^b^ meningitis aseptic, neuroborreliosis | 13 (1.5) | 0.05 (0.02–0.09) | 0.04 (0.01–0.12) |
| **Ear infections** | | Mastoiditis, otitis media, vestibular neuronitis, otitis externa | 8 (0.9) | 0.03  (0.01–0.06) | 0.03 (0.00–0.10) |
| **Hepatobiliary and spleen infections** | | Cholecystitis infective, hepatitis A, acute hepatitis C, biliary sepsis | 6 (0.7) | 0.02 (0.01–0.06) | 0.01 (0.00–0.08) |
| **Cardiac infections** | | Endocarditis, subacute endocarditis, viral pericarditis | 5 (0.6) | 0.02 (0.01–0.05) | 0.01 (0.00–0.08) |
| **Dental and oral infections** | | Gingivitis, tooth infection/abscess | 3 (0.3) | 0.01 (0.00–0.03) | 0.01 (0.00–0.08) |
| **Bone and joint infections** | | Osteomyelitis, bursitis infective, intervertebral discitis | 4 (0.5) | – | 0.06  (0.02–0.14) |
| **Breast infections** | | Mastitis, breast abscess | 4 (0.5) | 0.01  (0.00–0.03) | 0.04  (0.01–0.12) |
| **Eye and eyelid infections** | | Herpes ophthalmic^b^ | 1 (0.1) | – | 0.01 (0.00–0.08) |

^a^Only COVID-19 cases reported as of November 25, 2022; cells in gray highlight groups of SIs for which rates in patients with PMS had non-overlapping CIs with rates for patients with RMS, suggesting a significant difference between patients with PMS and RMS.

^b^Rate of serious herpes virus-associated infections = 0.03 per 100 PY (95% CI: 0.02–0.06); genital herpes (*n* = 1), genital herpes simplex
(*n* = 1), herpes ophthalmic (*n* = 1), herpes simplex (*n* = 1), herpes simplex meningitis (*n* = 1), herpes zoster (*n* = 2), varicella (*n* = 1), and varicella-zoster virus infection (*n* = 1).

CI, confidence interval; COVID-19, coronavirus disease 2019; GI, gastrointestinal; LRTI, lower respiratory tract infection; PMS, progressive multiple sclerosis; PY, patient years; RMS, relapsing multiple sclerosis; SI, serious infection; URTI, upper respiratory tract infection.

# Table S8. Serious infections of the central nervous system.

| **Reported term** | **Pathogen** | **Age at onset, years** | **Sex** | **Year of treatment** | **Type of MS** | **Intensity grade** | **Hospital duration, days** | **Outcome** | **EDSS, before/after event^a^** | **Action with OCR** |
| --- | --- | --- | --- | --- | --- | --- | --- | --- | --- | --- |
| **Meningitis bacterial** | Bacterial | 28 | F | 2 | RMS | Severe | 21 | Recovered/resolved | 0.0/0.0 | Not changed |
| **Meningitis bacterial** | Bacterial | 52 | F | 5 | PPMS | Life- threatening | 26 | Recovered/resolved | 2.5/3.5 | Not changed |
| **Meningitis viral** | Viral | 34 | M | 2 | RMS | Moderate | 5 | Recovered/resolved | 0.0/1.0 | Not changed |
| **Meningitis viral** | Viral | 41 | M | 5 | RMS | Severe | 4 | Recovered/resolved | 2.0/2.5 | Not changed |
| **Meningitis** | Unknown | 47 | M | 4 | RMS | Severe | 10 | Recovered/resolved | 4.0/4.0 | N/A^b^ |
| **Meningitis aseptic** | N/A | 41 | F | 7 | RMS | Severe | 13 | Recovered/resolved | 1.5/1.5 | Not changed |
| **Herpes simplex meningitis** | Viral | 47 | M | 8 | RMS | Severe | 21 | Recovered/resolved | 3.0/3.5 | Not changed |
| **Encephalitis** | Viral | 44 | F | 1 | PPMS | Severe | 8 | Recovered/resolved | 2.0/2.0 | Not changed |
| **Encephalitis** | Viral | 50 | F | 2 | RMS | Severe | – | Recovered/resolved | 2.0/2.0 | Not changed |
| **Encephalitis** | Bacterial | 47 | F | 7 | RMS | Life- threatening | 17 | Not recovered/not resolved | 1.5/3.0^c^ | Discontinued |
| **Encephalitis** | Unknown | 51 | F | 7 | RMS | Life- threatening | 15 | Not recovered/not resolved^d^ | 6.0/n.a. | Discontinued |
| **Encephalitis** | Unknown | 61 | F | 7 | PPMS | Death | 189 | Fatal | 4.0/n.a. | N/A |
| **Neuroborreliosis** | Bacterial | 47 | F | 9 | RMS | Life-  threatening | 21 | Recovered/resolved | 3.5/n.a. | Not changed |

^a^Nearest EDSS prior to the onset of the SI and after the resolution of the SI, or after the onset of the SI if resolution date was missing.
^b^N/A – event occurred after patient withdrew from treatment.
^c^An EDSS = 9.0 was subsequently reported in the safety follow-up. ^d^Patient died subsequently due to a COVID-19 infection.

Clinical cut-off date, November 2022.

COVID-19, coronavirus disease 2019; EDSS, Expanded Disability Status Scale; F, female; M, male; MS, multiple sclerosis; N/A, not applicable; n.a., not available; OCR, ocrelizumab;
PPMS, primary progressive multiple sclerosis; RMS, relapsing multiple sclerosis; SI, serious infection.

# Table S9. Patient demographics and characteristics of SIs during periods where IgG levels <5.65 g/L.^a^

**RMS**

| **Patient characteristics** | | | | **Event description** | | | | | | |  |
| --- | --- | --- | --- | --- | --- | --- | --- | --- | --- | --- | --- |
| **Sex** | **Age,^b^ years** | **EDSS^c^** | **Comorbidities** | | **Reported event^d^** | **Anatomical location** | **Intensity, grade** | **Duration, days** | **Outcome** | **Action with OCR** |  |
| F | 30 | 2.5 | Yes | | Anal abscess | Abdominal and GI | Severe | >28 | Recovered | Not changed |  |
| F | 35 | 1.0 | Yes | | Pyuria | Urinary tract | Moderate | ≤7 | Recovered | Not changed |  |
|  | 36 | 1.0 |  |  | Cellulitis | Skin | Severe | ≤7 | Recovered | Not changed |  |
| F | 39 | 5.5 | Yes | | Pneumonia | Respiratory tract | Severe | ≤7 | Recovered | Not changed |  |
| F | 43 | 2.0 | Yes | | Appendicitis | Abdominal and GI | Severe | ≤7 | Recovered | Not changed |  |
|  | 43 | 2.0 |  |  | Tubo-ovarian abscess | Reproductive tract | Severe | ≤7 | Recovered | Not changed |  |
| F | 43 | 1.0 | No | | Pneumonia | Respiratory tract | Severe | 15–28 | Recovered | Not changed |  |
| F | 47 | 4.5 | Yes | | Sepsis | Sepsis | Life-threatening | 15–28 | Recovered | Not changed |  |
| F | 49 | 3.0 | Yes | | Pneumonia hemophilus | Respiratory tract | Moderate | >28 | Recovered | Not changed |  |
| F | 50 | 2.0 | Yes | | Tooth abscess | Dental and oral | Severe | ≤7 | Recovered | Not changed |  |
| F | 53 | 4.0 | Yes | | Pyelonephritis | Urinary tract | Severe | 8–14 | Recovered | Not changed |  |
|  | 54 | 4.5 |  |  | Pseudomonas infection | Skin | Life-threatening | ≤7 | Recovered | Not changed |  |
| F | 55 | 3.0 | Yes | | Chronic rhinosinusitis | Respiratory tract | Severe | >28 | Recovered | Withdrawn |  |
| F | 55 | 1.5 | No | | Salpingitis | Reproductive tract | Severe | ≤7 | Recovered | Not changed |  |
| F | 64 | 4.5 | No | | Pneumonia | Respiratory tract | Life-threatening | 8–14 | Recovered | Not changed |  |
| M | 42 | 1.0 | No | | Pneumonia bacterial | Respiratory tract | Severe | >28 | Recovered | Not changed |  |
| M | 42 | 1.0 | Yes | | Pneumonia | Respiratory tract | Moderate | 15–28 | Recovered | Not changed |  |
| M | 44 | 6.0 | Yes | | Urinary tract infection | Urinary tract | Moderate | 8–14 | Recovered | Not changed |  |
| M | 47 | 4.5 | Yes | | Acute sinusitis | Respiratory tract | Severe | 15–28 | Recovered | Not changed |  |
|  | 47 | 4.5 |  |  | Acute sinusitis | Respiratory tract | Severe | 8–14 | Recovered with sequelae | Not changed |  |
| M | 52 | 1.5 | No | | Endocarditis | Cardiac infections | Life-threatening | >28 | Recovered | Withdrawn |  |
| M | 58 | 1.5 | Yes | | Device-related infection | **–** | Severe | 15–28 | Recovered | Not changed |  |
| M | 58 | 4.0 | Yes | | Pneumonia | Respiratory tract | Severe | >28 | Recovered | Withdrawn |  |
| M | 62 | 4.0 | Yes | | Bronchitis | Respiratory tract | Moderate | 8–14 | Recovered | Not changed |  |

**PPMS**

| **Patient characteristics** | | | | **Event description** | | | | | | |  |
| --- | --- | --- | --- | --- | --- | --- | --- | --- | --- | --- | --- |
| **Sex** | **Age,^b^ years** | **EDSS^c^** | **Comorbidities** | **Reported event^d^** | **Anatomical location** | **Intensity, grade** | **Duration, days** | **Outcome** | **Action with OCR** |  |  |
| F | 46 | 7.0 | Yes | Sepsis | Sepsis | Life-threatening | 8–14 | Recovered | Not changed |  | |
|  | 49 | 7.5 |  | Sepsis | Sepsis | Moderate | ≤7 | Recovered | Not changed |  | |
| F | 51 | 6.5 | Yes | Bronchitis | Respiratory tract | Moderate | 8–14 | Recovered | Not changed |  | |
| F | 53 | 7.0 | No | Mastitis | Breast | Severe | >28 | Recovered | Not changed |  | |
|  | 54 | 7.5 |  | Mastitis | Breast | Life-threatening | >28 | Recovered | Not changed |  | |
| F | 58 | 8.5 | No | Pyelonephritis | Urinary tract | Moderate | ≤7 | Recovered | Not changed |  | |
|  | 59 | 7.5 |  | Pyelonephritis | Urinary tract | Severe | 15–28 | Recovered | Not changed |  | |
|  | 60 | 9.0 |  | Pneumonia | Respiratory tract | Severe | 15–28 | Recovered | Not changed |  | |
| F | 59 | 9.0 | Yes | Urinary tract Infection | Urinary tract | Severe | ≤7 | Recovered | Not changed |  | |
| F | 61 | 7.0 | Yes | Urinary tract infection | Urinary tract | Severe | 15–28 | Recovered | Not changed |  | |
|  | 62 | 7.0 |  | Post-procedural sepsis | Sepsis | Life-threatening | 8–14 | Recovered | Not changed |  | |
| M | 39 | 6.0 | Yes | Cellulitis | Skin | Life-threatening | 8–14 | Recovered | Not changed |  | |
| M | 53 | 6.0 | Yes | Enteritis infectious | Abdominal and GI | Moderate | >28 | Recovered | Temporary interruption |  | |
| M | 54 | 6.0 | Yes | Bronchiolitis | Respiratory tract | Severe | ≤7 | Recovered | Not changed |  | |
| M | 61 | 5.5 | Yes | Cellulitis | Skin | Severe | – | Not recovered | N/A |  | |
| M | 61 | 7.0 | Yes | Pneumonia Escherichia | Respiratory tract | Severe | >28 | Recovered | Not changed |  | |

^a^5.65 g/L corresponds to the lower limit of normal of the assay used in the central lab for measuring immunoglobulin levels.

^b^Age is listed as that at the onset of the SI.
^c^EDSS recorded as last known value prior to the SI.
^d^Investigator text for SIs encoded using MedDRA version 23.1 (known as preferred term).

A total of 21 cases of COVID-19–related SIs were reported in 19 patients with IgG<LLN; of which, 13 cases recovered/recovered without sequelae, two cases recovered/resolved with sequelae and six fatalities were recorded.

COVID-19, coronavirus disease 2019; EDSS, Expanded Disability Status Scale; F, female; GI, gastrointestinal; Ig, immunoglobulin; LLN, lower limit of normal; M, male; MedDRA, Medical Dictionary for Regulatory Activities; N/A, not applicable; OCR, ocrelizumab; PPMS, primary progressive multiple sclerosis; Pt, patient; RMS, relapsing multiple sclerosis; SI, serious infection.

# Table S10. Demographic and clinical characteristics of patients who discontinued (discontinuers) or completed (completers) in OPERA or ORATORIO and respective OLEs.

|  | **OPERA** | | **ORATORIO** | |
| --- | --- | --- | --- | --- |
| **Characteristic, *n* (%) except where specified** | **Completers**  **(*n* = 872)** | **Discontinuers**  **(*n* = 576)** | **Completers**  **(*n* = 368)** | **Discontinuers**  **(*n* = 276)** |
| **Median time on treatment** | 9.2 | 4.9 | 10.1 | 5.7 |
| **Age at OCR start,^a^ years** |  |  |  |  |
| Mean (SD) | 38.0 (8.9) | 38.2 (9.8) | 46.0 (8.1) | 45.5 (8.1) |
| Median | 38.0 | 38.0 | 48.0 | 47.0 |
| Min–Max | 18.0–57.0 | 18.0–58.0 | 23.0–59.0 | 20.0–59.0 |
| **Age at last known follow-up^b^** |  |  |  |  |
| <40 years | 189 (21.7) | 224 (38.9) | 17 (4.6) | 29 (10.5) |
| 40–59 years | 595 (68.2) | 316 (54.9) | 228 (62.0) | 207 (75.0) |
| ≥60 years | 88 (10.1) | 36 (6.3) | 123 (33.4) | 40 (14.5) |
| **Sex** |  |  |  |  |
| Female | 550 (63.1) | 399 (69.3) | 193 (52.4) | 127 (46.0) |
| Male | 322 (36.9) | 177 (30.7) | 175 (47.6) | 149 (54.0) |
| **BMI (kg/m^2^) at baseline** |  |  |  |  |
| Underweight <18.5 | 35 (4.0) | 21 (3.6) | 13 (3.5) | 14 (5.1) |
| Normal weight 18.5 to <25.0 | 419 (48.1) | 258 (44.8) | 196 (53.3) | 149 (54.0) |
| Overweight 25.0 to <30.0 | 220 (25.2) | 169 (29.3) | 98 (26.6) | 76 (27.5) |
| Obese ≥30.0 | 186 (21.3) | 126 (21.9) | 60 (16.3) | 37 (13.4) |
| **Comorbidities at OCR start^a^** |  |  |  |  |
| None | 409 (46.9) | 256 (44.4) | 156 (42.4) | 102 (37.0) |
| 1 | 295 (33.8) | 193 (33.5) | 113 (30.7) | 85 (30.8) |
| ≥2 | 168 (19.3) | 127 (22.0) | 99 (26.9) | 89 (32.2) |
| **Comorbidities at last known follow-up^b^** |  |  |  |  |
| None | 295 (33.8) | 196 (34.0) | 106 (28.8) | 83 (30.1) |
| 1 | 265 (30.4) | 193 (33.5) | 90 (24.5) | 63 (22.8) |
| ≥2 | 312 (35.8) | 187 (32.5) | 172 (46.7) | 130 (47.1) |
| **EDSS at OCR start^a^** |  |  |  |  |
| <3.0 | 511 (58.6) | 299 (51.9) | 10 (2.7) | 2 (0.7) |
| 3.0–6.0 | 356 (40.8) | 269 (46.7) | 319 (86.7) | 210 (76.1) |
| >6.0 | 5 (0.6) | 8 (1.4) | 39 (10.6) | 63 (22.8) |
| **EDSS at last known follow-up^b^** |  |  |  |  |
| <3.0 | 465 (53.3) | 262 (45.5) | 14 (3.8) | 9 (3.3) |
| 3.0–6.0 | 368 (42.2) | 280 (48.6) | 218 (59.2) | 135 (48.9) |
| >6.0 | 39 (4.5) | 34 (5.9) | 136 (37.0) | 132 (47.8) |
| **Disease duration since symptom onset** |  |  |  |  |
| <5 years | 399 (45.8) | 259 (45.0) | 110 (29.9) | 80 (29.0) |
| 5–10 years | 243 (27.9) | 162 (28.1) | 168 (45.7) | 120 (43.5) |
| >10 years | 230 (26.4) | 155 (26.9) | 80 (21.7) | 67 (24.3) |
| **Relapses in year before randomization** |  |  |  |  |
| No | 343 (39.3) | 190 (33.0) | – | – |
| Yes | 529 (60.7) | 386 (67.0) | – | – |

^a^In the year prior to OCR start (for patients switching from interferon or placebo, this corresponds to the last year of the double-blind period).

^b^Last known follow-up defined as of December 31, 2022, the final date of study discontinuation or completion.

BMI, body mass index; EDSS, Expanded Disability Status Scale; Max, maximum; Min, minimum; OCR, ocrelizumab; OLE, open-label extension; SD, standard deviation.

# Supplementary Table References

1. U.S. Food & Drug Administration. What is a serious adverse event?, <https://www.fda.gov/safety/reporting-serious-problems-fda/what-serious-adverse-event>.
(2016, accessed 9 August 2022).

2. National Cancer Institute. Common Terminology Criteria for Adverse Events (CTCAE), <https://evs.nci.nih.gov/ftp1/CTCAE/CTCAE_4.03/Documentation/CTCAE_Governance_2010-03-11.pdf>. (2010, accessed 29 October 2021).

3. Charlson ME, Pompei P, Ales KL, *et al*. A new method of classifying prognostic comorbidity in longitudinal studies: development and validation. *J Chronic Dis* 1987; 40: 373–383.

4. Elixhauser A, Steiner C, Harris DR, *et al*. Comorbidity measures for use with administrative data. *Med Care* 1998; 36: 8–27.

5. Andersson NW, Goodwin RD, Okkels N, *et al*. Depression and the risk of severe infections: prospective analyses on a nationwide representative sample. *Int J Epidemiol* 2016; 45: 131–139.
